# Effects of monoglyceride blend on systemic and intestinal immune responses, and gut health of weaned pigs experimentally infected with a pathogenic *Escherichia coli*

**DOI:** 10.1186/s40104-024-01103-7

**Published:** 2024-10-13

**Authors:** Sangwoo Park, Shuhan Sun, Lauren Kovanda, Adebayo O. Sokale, Adriana Barri, Kwangwook Kim, Xunde Li, Yanhong Liu

**Affiliations:** 1 Department of Animal Science, University of California, Davis, CA 95616 USA; 2 BASF Corporation, Florham Park, 07932 USA; 3 BASF SE, Lampertheim, Germany; 4 Department of Animal Science, Michigan State University, East Lansing, MI 48824 USA; 5 School of Veterinary Medicine, University of California, Davis, CA 95616 USA

**Keywords:** Diarrhea, Enterotoxigenic *Escherichia coli*, Gut health, Monoglycerides, Systemic immunity, Weaned pigs

## Abstract

**Background:**

Monoglycerides have emerged as a promising alternative to conventional practices due to their biological activities, including antimicrobial properties. However, few studies have assessed the efficacy of monoglyceride blend on weaned pigs and their impacts on performance, immune response, and gut health using a disease challenge model. Therefore, this study aimed to investigate the effects of dietary monoglycerides of short- and medium-chain fatty acids on the immunity and gut health of weaned pigs experimentally infected with an enterotoxigenic *Escherichia coli* F18.

**Results:**

Pigs supplemented with high-dose zinc oxide (ZNO) had greater (*P* < 0.05) growth performance than other treatments, but no difference was observed in average daily feed intake between ZNO and monoglycerides groups during the post-challenge period. Pigs in ZNO and antibiotic groups had lower (*P* < 0.05) severity of diarrhea than control, but the severity of diarrhea was not different between antibiotic and monoglycerides groups. Pigs fed with monoglycerides or ZNO had lower (*P* < 0.05) serum haptoglobin on d 2 or 5 post-inoculation than control. Pigs in ZNO had greater (*P* < 0.05) goblet cell numbers per villus, villus area and height, and villus height:crypt depth ratio (VH:CD) in duodenum on d 5 post-inoculation than pigs in other treatments. Pigs supplemented with monoglycerides, ZNO, or antibiotics had reduced (*P* < 0.05) ileal crypt depth compared with control on d 5 post-inoculation, contributing to the increase (*P* = 0.06) in VH:CD. Consistently, pigs in ZNO expressed the lowest (*P* < 0.05) *TNFa*, *IL6*, *IL10*, *IL12*, *IL1A*, *IL1B*, and *PTGS2* in ileal mucosa on d 5 post-inoculation, and no difference was observed in the expression of those genes between ZNO and monoglycerides. Supplementation of ZNO and antibiotic had significant impacts on metabolic pathways in the serum compared with control, particularly on carbohydrate and amino acid metabolism, while limited impacts on serum metabolites were observed in monoglycerides group when compared with control.

**Conclusions:**

The results suggest that supplementation of monoglyceride blend may enhance disease resistance of weaned pigs by alleviating the severity of diarrhea and mitigating intestinal and systemic inflammation, although the effectiveness may not be comparable to high-dose zinc oxide.

**Supplementary Information:**

The online version contains supplementary material available at 10.1186/s40104-024-01103-7.

## Background

Weaning piglets, the process of separating them from their mother, exposes them to nutritional, physiological, and environmental challenges [1–3]. These weaning stressors impair intestinal barrier function and induce intestinal and systemic inflammation, in addition to the typically occurring decrease in feed intake [4, 5]. The compromised intestinal barrier increases the risk of external factors (e.g., toxins, antigens, and pathogens) entering the body, making piglets vulnerable to enteric diseases [6, 7]. Post-weaning diarrhea, caused by the infection of enterotoxigenic *Escherichia coli* (ETEC) F18, is one of the common problems in young pigs [8, 9]. This disease is characterized by watery diarrhea and deterioration of intestinal health, causing tremendous economic losses in swine production due to growth lag, morbidity, cost of medication, and mortality [10–13]. In-feed antibiotics or pharmacological doses of zinc oxide (2,000–3,000 mg/kg) have been widely applied to nursery diets for controlling post-weaning diarrhea and promoting animal health and growth [14–16]. However, along with the increased public health concern regarding antimicrobial resistance [17–22], the use of antibiotics for growth promoting purposes in animal production has been restricted since 2017 in the United States [23]. Furthermore, considering sustainable animal agriculture, it is noteworthy that Europe not only banned the use of pharmacological doses of zinc oxide but also limited dietary zinc oxide supplementation to 150 mg/kg [24–26]. Hence, alternative practices, including animal management and nutrition interventions, are needed to promote animal health and welfare, as increased morbidity and economic losses due to the constraints of conventional practices are inevitable.

Numerous nutritional interventions (e.g., exogenous enzymes, bioactive compounds derived from animals or plants, microbiome modulators) have been investigated and adopted in the swine industry to address the emergence of the post-antibiotic era [27, 28]. One promising alternative is a group of products based on organic acids, specifically short-chain fatty acids (SCFA; less than 6 carbons) or medium-chain fatty acids (MCFA; 6–12 carbons). Research has shown that SCFA and MCFA have strong antibacterial activity [29–31]. In addition, they also exhibit various biological activities in pigs [32–34], including beneficial effects on growth performance, intestinal physiology, and immunity, making them more than just an energy source. However, the effectiveness of supplementing organic fatty acids is often hindered by limiting factors such as unpalatable flavor and losses prior to reaching the lower gastrointestinal tract [35, 36]. In this respect, monoglycerides, composed of fatty acid esterified to glycerol, may address the limitations due to the two criteria: (1) they are relatively easy to handle; and (2) they allow active substances to be gradually released throughout the intestine [37]. Moreover, in vitro antimicrobial activity against a wide range of pathogenic bacteria was observed in glycerol esters derived from SCFA and MCFA [30, 38–41]. There is growing interest in monoglycerides as antibacterial lipids in nutrition and health. Their physiological activities have been extensively studied in poultry [42–44], however, limited research has been reported on the efficacy of monoglycerides in weaned pigs using disease models. Therefore, the objective of this study was to investigate the influence of dietary supplementation of a monoglyceride blend on growth performance, intestinal health, and systemic immunity of weaned pigs experimentally infected with ETEC F18.

## Materials and methods

### Animals, housing, experimental design, and diet

Sixty weaned pigs with 28 barrows and 32 gilts (average body weight [BW] = 6.49 ± 0.74 kg; around 21 to 24 d old) were obtained from the Swine Teaching and Research Center at the University of California, Davis, USA. The sows and piglets used in this experiment did not receive *Escherichia coli* vaccines, antibiotic injections, or antibiotics in creep feed. Before weaning, feces were collected from sows and all their piglets destined for this study to verify the absence of β-hemolytic *Escherichia coli*. The ETEC F18 receptor status was also tested by polymerase chain reaction (PCR)-restriction fragment length polymorphism [45], and piglets susceptible to ETEC F18 were selected for this study. After weaning, all pigs were randomly assigned to one of the four dietary treatments (15 replicates/treatment) in a randomized complete block design with BW within sex and litter as the block and pig as the experimental unit. Pigs were housed in individual pens (0.61 m × 1.22 m) for 28 d, including 7 d before and 21 d after the first ETEC challenge. All piglets had free access to feed and water. The light was on at 07:30 h and off at 19:30 h daily in the environmental control unit.

The four dietary treatments included: (1) a corn-soybean meal-based basal diet (control); (2) the basal diet with 0.3% monoglyceride blend (BalanGut™ LS L; BASF SE, Ludwigshafen, Germany) of butyric, caprylic, and capric acids; (3) the basal diet with 3,000 mg/kg of zinc oxide (ZNO); (4) the basal diet with 50 mg/kg of carbadox (antibiotic). A 2-phase feeding program was used with the first two weeks as phase 1 and the last two weeks as phase 2 (Table 1). Spray-dried plasma, antibiotics, and high levels of zinc oxide exceeding recommendation and normal practice were not included in basal diet. All diets were formulated to meet pig nutritional requirements [46] and provided as mash form throughout the experiment.

**Table 1:** Ingredient compositions of experimental diets^a^

Ingredient, %	Control (phase 1)	Control (phase 2)
Corn	44.41	57.27
Dried whey	15.00	10.00
Soybean meal	18.00	22.00
Fish meal	10.00	7.00
Lactose	6.00	-
Soy protein concentrate	3.00	-
Soybean oil	2.00	2.00
Limestone	0.56	0.70
L-Lysine·HCl	0.21	0.23
DL-Methionine	0.08	0.05
L-Threonine	0.04	0.05
Salt	0.40	0.40
Vit-mineral (Sow 6)^b^	0.30	0.30
Total	100.00	100.00
Calculated energy and nutrient		
Metabolizable energy, kcal/kg	3,463	3,429
Net energy, kcal/kg	2,601	2,575
Crude protein, %	22.27	20.80
Arg^c^, %	1.23	1.15
His^c^, %	0.49	0.47
Ile^c^, %	0.83	0.76
Leu^c^, %	1.62	1.55
Lys^c^, %	1.35	1.23
Met^c^, %	0.45	0.39
Thr^c^, %	0.79	0.73
Trp^c^, %	0.23	0.21
Val^c^, %	0.91	0.84
Met + Cys^c^, %	0.74	0.68
Phe + Tyr^c^, %	1.45	1.38
Ca, %	0.80	0.70
Total P, %	0.68	0.59
Digestible P, %	0.47	0.37

^a^In each phase, three additional diets were formulated by adding 0.3% monoglyceride blend, 3,000 mg/kg zinc oxide, or 50 mg/kg carbadox to the control diet, respectively

^b^Provided by the United Animal Health (Sheridan, IN, USA)

^c^Amino acids were indicated as standardized ileal digestible amino acids

After 7 days of adaptation, all pigs were orally inoculated with 3 mL of ETEC F18 for three consecutive days from d 0 post-inoculation (PI). The ETEC F18 was originally isolated from a field disease outbreak by the University of Montreal (isolate number: ECL22131). The ETEC F18 expresses heat-labile toxin and heat-stable toxins a and b. The inoculums were prepared at 10^10^ colony-forming units per 3 mL dose in phosphate buffered saline. This dose caused mild diarrhea in the current study, consistent with our previously published research [47–49].

### Clinical observations and sample collections

The procedures of this experiment were adapted from previous research [47, 50–52]. Clinical observations (fecal score and alertness score) were recorded twice daily throughout the study. The fecal score of each pig was assessed each day visually by two independent evaluators, with the score ranging from 1 to 5 (1 = normal feces, 2 = moist feces, 3 = mild diarrhea, 4 = severe diarrhea, and 5 = watery diarrhea). The frequency of diarrhea was calculated as the percentage of the pig days with fecal score of 3 or greater, as well as calculated as the percentage of the pig days with fecal score of 4 or greater. Alertness was scored from 1 to 3 (1 = normal, 2 = slightly depressed or listless, and 3 = severely depressed or recumbent). Scores for alertness did not exceed two throughout the experiment (data not shown).

Pigs were weighed on weaning day (d −7; initial BW), d 0 (before first inoculation), 5, 14, and 21 PI. Feed intake was recorded throughout the study. Average daily gain (ADG), average daily feed intake (ADFI), and feed efficiency (gain:feed ratio) were calculated for each period. Fecal samples were collected from the rectum of all pigs throughout the experiment using a cotton swab on d −7, 2, 5, 7, 10, 14, and 21 PI to test β-hemolytic coliforms and the percentage of β-hemolytic coliforms to total coliforms [47, 50–52]. Blood samples were collected from the jugular vein of all pigs before ETEC challenge (d 0), and on d 2, 5, 14, and 21 PI to collect serum samples, which were stored at − 80°C until further analysis.

Twenty-four pigs (6 pigs/treatment, 3 barrows and 3 gilts) were euthanized on d 5 PI near the peak of ETEC infection, and the remaining pigs were euthanized at the end of the experiment (d 21 PI). Before euthanization, pigs were anesthetized with 1 mL mixture of 100 mg Telazol, 50 mg ketamine, and 50 mg xylazine (2:1:1) by intramuscular injection. After anesthesia, intracardiac injection with 78 mg Fatal-Plus solution (sodium pentobarbital, MWI Animal Health, Visalia, CA, USA) per 1 kg of BW was used to euthanize each pig. Intestinal mucosa samples were collected from jejunum and ileum, snap-frozen in liquid nitrogen, and then stored at −80 °C for gene expression analysis. Three 4-cm segments from the duodenum, the middle of the jejunum, and the ileum (10 cm close to the ileocecal junction) were collected and fixed in 10% neutral buffered formalin for intestinal morphology analysis.

### Detection of β-hemolytic coliforms

Briefly, fecal samples were plated on Columbia Blood Agar with 5% sheep blood to identify hemolytic coliforms, which can lyse red blood cells surrounding the colony. Fecal samples were also plated on MacConkey agar to enumerate total coliforms. Hemolytic colonies from the blood agar were sub-cultured on MacConkey agar to confirm that they were lactose-fermenting bacteria and flat pink colonies. All plates were incubated at 37 °C for 24 h in an air incubator. Populations of both total coliforms and β-hemolytic coliforms on blood agar were visually scored from 0 to 8 (0 = no bacterial growth, 8 = very heavy bacterial growth). The ratio of scores of β-hemolytic coliforms to total coliforms was calculated.

### Measurements of serum cytokine and acute phase proteins

Serum samples were analyzed for tumor necrosis factor-α (TNF-α; R&D Systems Inc., Minneapolis, MN, USA), C-reactive protein (CRP; R&D Systems Inc., Minneapolis, MN, USA), and haptoglobin (Aviva Systems Biology, San Diego, CA, USA) using porcine-specific enzyme-linked immunosorbent assay kits following the manufacturer’s procedures. All samples, including standards, were analyzed in duplicate. The intensity of the color was measured at 450 nm with the correction wavelength set at 530 nm using a plate reader (BioTek Instruments, Inc., Winooski, VT, USA). The intra-assay coefficients of variation for TNF-α, CRP, and haptoglobin were less than 7%. The inter-assay coefficients of variation for TNF-α, CRP, and haptoglobin were less than 10%. The concentrations of each analyte in the tested samples were calculated based on a standard curve.

### Intestinal morphology

Fixed intestinal tissues were embedded in paraffin, sectioned at 5 μm, and stained with hematoxylin and eosin. The slides were photographed by an Olympus BX51 microscope at 10× magnification, and all measurements were conducted in the image processing and analysis software (Image J, NIH). Ten straight and integrated villi and their associated crypts and surrounding areas were selected to analyze villus height (VH), area, and width; crypt depth (CD) and width; and goblet cell number per villus as described in previous studies [52, 53].

### Immunohistochemistry

The immunohistochemistry procedures were based on previous research [47, 54]. Briefly, the embedded ileal tissues were sectioned at 5 μm and placed on the microslides. The slides were incubated with 5 µg/mL porcine neutrophil-specific antibody PM1 (BMA Biomedicals, Augst, Switzerland) or 0.4 µg/mL porcine macrophage-specific antibody MAC387 (Thermo Scientific, Waltham, MA, USA). Antibody binding was visualized by using the avidin-biotin complex, and the diaminobenzidine chromogen (Vector Laboratories, Inc., CA, USA). Hematoxylin was applied as a counter stain. Slides incubated without the primary antibodies but with PBS were used as negative controls. Images were captured by an Olympus BX51 microscope at 10× magnification, and all measurements were analyzed by Image J software. Eight straight and integrated ileal villi were selected for measurement. The unit was the number of cells/mm^2^.

### Intestinal barrier and innate immunity

Jejunal and ileal mucosa samples were analyzed for gene expression by quantitative real-time PCR (qRT-PCR). Briefly, approximately 100 mg of mucosa sample was homogenized using TRIzol reagent (Invitrogen; Thermo Fisher Scientific, Inc., Waltham, MA, USA). Then, total ribonucleic acid (RNA) was extracted following RNA extraction procedural guidelines provided by the reagent manufacturer. The quality and quantity of RNA were evaluated using a Thermo Scientific NanoDrop 2000 Spectrophotometer (Thermo Fisher Scientific, Inc., Waltham, MA, USA). The complementary DNA (cDNA) was produced from 1 µg of total RNA per sample using the High-Capacity cDNA Reverse Transcription Kit (Applied Biosystems; Thermo Fisher Scientific, Inc., Waltham, MA, USA) in a total volume of 20 µL. The mRNA expression of Mucin 2 (*MUC2*), Claudin-1 (*CLDN1*), Zonula occludens-1 (*ZO-1*), and Occludin (*OCLN*) in jejunal mucosa and the mRNA expression of Tumor necrosis factor-alpha (*TNFa*), Interleukin 6 (*IL6*), Interleukin 7 (*IL7*), Interleukin 10 (*IL10*), Interleukin 12 (*IL12*), Interleukin-1 alpha (*IL1A*), Interleukin-1 beta (*IL1B*), *MUC2*, and Prostaglandin-endoperoxide synthase 2 (*PTGS2*) in ileal mucosa were analyzed. Data normalization was accomplished using 18S ribosomal RNA as a housekeeping gene. Primers were designed based on published literature and commercially synthesized by Integrated DNA Technologies, Coralville, IA, USA. All primers were verified prior to qRT-PCR (Table S1). The qRT-PCR reaction conditions followed the published research [55]. The 2^−ΔΔCT^ method was used to analyze the relative expression of genes compared to control [56].

### Untargeted metabolomics analysis

The untargeted metabolomics analysis was performed by the NIH West Coast Metabolomics Center at the University of California, Davis, using gas chromatography (Agilent 6890 gas chromatograph controlled using Leco ChromaTOF software version 2.32, Agilent, Santa Clara, CA, USA) coupled with time-of-flight mass spectrometry (GC/TOF-MS) (Leco Pegasus IV time-of-flight mass spectrometer controlled using Leco ChromaTOF software version 2.32, Leco, St. Joseph, MI, USA). Metabolite extraction was performed following procedures previously described by Fiehn et al. [57]. Briefly, frozen serum samples (approximately 30 µL) were homogenized using a Retsch ball mill (Retsch, Newtown, PA, USA) for 30 s at 25 times/s. After homogenization, a prechilled (−20 °C) extraction solution (isopropanol/acetonitrile/water at the volume ratio 3:3:2, degassed with liquid nitrogen) was added at a volume of 1 mL/20 mg of sample. Samples were then vortexed and shaken for metabolite extraction. After centrifugation at 12,800 × *g* for 2 min, the supernatant was collected and divided into two equal aliquots and concentrated at room temperature for 4 h in a cold-trap vacuum concentrator (Labconco Centrivap, Kansas City, MO, USA). To separate complex lipids and waxes, the residue was re-suspended in 500 µL of 50% aqueous acetonitrile and centrifuged at 12,800 × *g* for 2 min. The resultant supernatant was collected and concentrated in the vacuum concentrator. Dried sample extracts were derivatized and mixed with internal retention index markers (fatty acid methyl esters with the chain length of C8 to C30). The samples were injected for GC/TOF analysis, and all samples were analyzed in a single batch. Data acquisition by mass spectrometry and mass calibration using FC43 (perfluorotributylamine) before starting analysis sequences. Metabolite identifications were performed based on the two parameters: (1) Retention index window ± 2,000 U (around ± 2 s retention time deviation), and (2) Mass spectral similarity plus additional confidence criteria as detailed below. A detailed methodology for data acquisition and metabolite identification was described in a previously published article by Fiehn et al. [57].

### Statistical analysis

The normality of data was verified and outliers were identified using the UNIVARIATE procedure (SAS Institute Inc., Cary, NC, USA). Outliers were identified and removed as values that deviated from the treatment mean by more than 3 times the interquartile range. All data except frequency of diarrhea and metabolomics were analyzed by ANOVA using the PROC MIXED of SAS (SAS Institute Inc., Cary, NC, USA) in a randomized complete block design with the pig as the experimental unit. The statistical model included diet as the main effect and block as random effect. Treatment means were separated by using the LSMEANS statement and the PDIFF option of PROC MIXED. The Chi-square test was used for analyzing the frequency of diarrhea. Statistical significance and tendency were considered at *P* < 0.05 and 0.05 ≤ *P* < 0.10, respectively.

The metabolomics data were analyzed using different modules of a web-based platform, MetaboAnalyst 5.0 (https://www.metaboanalyst.ca) [58]. Data were filtered for peaks with detection rates less than 30% of missing abundances and normalized using logarithmic transformation and auto-scaling. Mass univariate analysis was performed using one-way ANOVA followed by Fisher’s least significant difference test (adjusted *P* ≤ 0.05). Fold change analysis and *t*-tests were also conducted to determine the fold change and significance of each identified metabolite. Statistical significance was declared at a false discovery rate (FDR, Benjamini and Hochberg correction; q) < 0.2 and fold change > 2.0. Partial least squares discriminant analysis (PLS-DA) was carried out to further identify discriminative variables (metabolites) among the treatment groups. Pathway analysis and metabolite set enrichment analysis were performed on identified metabolites that had a Variable Importance in Projection (VIP) score > 1. The pathway with a *P*-value less than 0.05, as well as an impact value greater than 0.1, was defined as a significant impact pathway.

## Results

### Growth performance, diarrhea, β-hemolytic coliforms

There were no significant differences in the initial (d −7) and d 0 BW of pigs among dietary treatments (Table 2). In comparison to control and antibiotic groups, supplementation of monoglycerides did not affect BW, ADG, and ADFI throughout the experiment. Pigs supplemented with ZNO had greater (*P* < 0.05) BW on d 5, 14, and 21 PI, increased (*P* < 0.05) ADG from d 0 to 5 PI, d 0 to 14 PI, and d 0 to 21 PI, and enhanced (*P* < 0.05) ADFI from d 0 to 14 PI and d 0 to 21 PI than the other treatments. However, the ADFI from d 0 to 21 PI was not different between ZNO and monoglycerides groups. Pigs supplemented with ZNO had greater (*P* < 0.01) gain:feed ratio from d 0 to 5 PI compared with the other treatments, but the difference did not persist throughout the post-challenge period. The gain:feed ratio on d 0 to 21 PI was lower (*P* < 0.05) in monoglycerides than in control and antibiotic groups, but did not differ from ZNO group.

**Table 2:** Growth performance of enterotoxigenic *Escherichia coli* F18-challenged weaned pigs fed experimental diets

Item^c^	Control	Monoglycerides	ZNO^d^	Antibiotic	SEM	*P*-value
BW, kg						
d −7	6.50	6.47	6.51	6.48	0.19	0.95
d 0	7.44	7.46	7.81	7.43	0.23	0.12
d 5 PI	7.56^b^	7.38^b^	9.25^a^	7.95^b^	0.34	< 0.01
d 14 PI^e^	12.88^b^	12.56^b^	14.71^a^	12.77^b^	0.43	< 0.05
d 21 PI^e^	17.43^b^	17.00^b^	19.14^a^	17.33^b^	0.52	< 0.05
ADG, g						
d −7 to 0	154	145	186	135	20.97	0.30
d 0 to 5 PI	38^b^	44^b^	287^a^	104^b^	34.14	< 0.01
d 0 to 14 PI^e^	346^b^	330^b^	452^a^	362^b^	26.98	< 0.05
d 0 to 21 PI^e^	470^b^	431^b^	526^a^	457^b^	15.29	< 0.01
ADFI, g						
d −7 to 0	271	267	278	273	28.71	0.98
d 0 to 5 PI	353	376	451	405	28.64	0.14
d 0 to 14 PI^e^	553^b^	607^b^	718^a^	577^b^	25.93	< 0.01
d 0 to 21 PI^e^	680^b^	741^ab^	826^a^	719^b^	32.32	< 0.05
Gain:Feed						
d −7 to 0	0.55	0.54	0.67	0.48	0.054	0.10
d 0 to 5 PI	0.10^b^	0.11^b^	0.60^a^	0.23^b^	0.069	< 0.01
d 0 to 14 PI^e^	0.62	0.55	0.63	0.61	0.029	0.25
d 0 to 21 PI^e^	0.66^a^	0.58^b^	0.62^ab^	0.64^a^	0.014	< 0.05

^a,b^Means without a common superscript are different (*P* < 0.05)

^c^*BW* Body weight, *ADG* Average daily gain, *ADFI* Average daily feed intake, *PI* Post-inoculation. Each least squares mean represents 14–15 observations

^d^*ZNO* High-dose zinc oxide

^e^Each least squares mean represents 8–9 observations

**Fig. 1:**
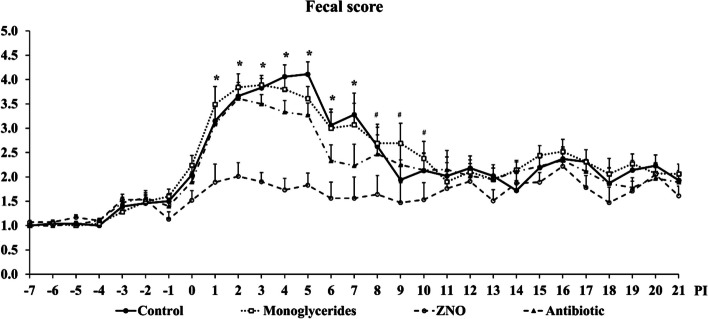
Daily fecal score of enterotoxigenic *Escherichia coli* F18-challenged weaned pigs fed diets supplemented with monoglycerides, high-dose zinc oxide (ZNO), or antibiotic. Fecal score = 1, normal feces; 2, moist feces; 3, mild diarrhea; 4, severe diarrhea; 5, watery diarrhea. ^*^P < 0.05, indicating fecal scores were significantly different among treatments. ^#^*P* < 0.10, indicating fecal scores tended to different among treatments. Each least squares mean represents 14–15 observations before d 5 post-inoculation (PI) and each least squares mean represents 8–9 observations after d 5 PI

**Fig. 2:**
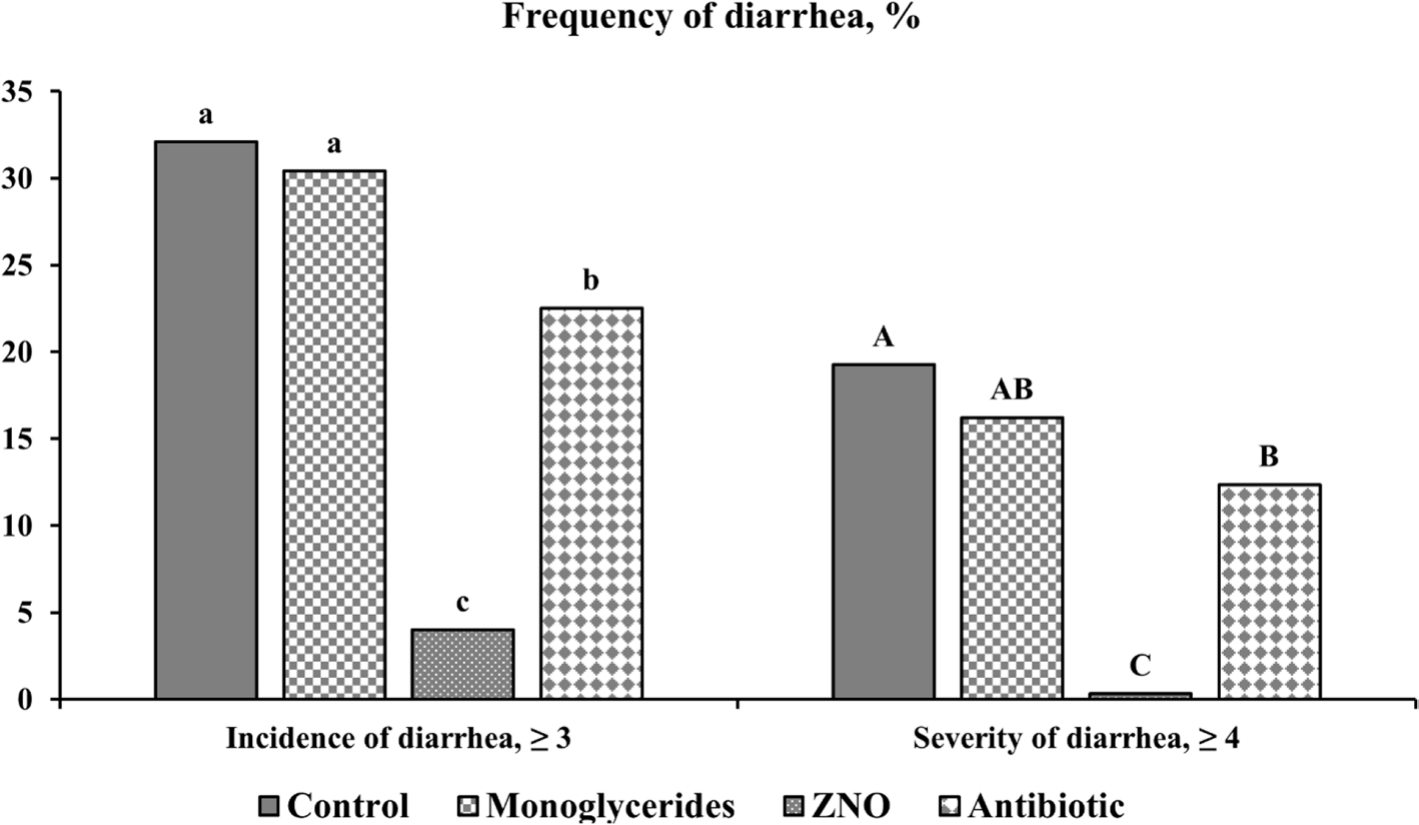
Frequency of diarrhea (overall period) of enterotoxigenic *Escherichia coli* F18-challenged weaned pigs fed diets supplemented with monoglycerides, high-dose zinc oxide (ZNO), or antibiotic. Frequency of diarrhea was calculated as the percentage of pig days with fecal score ≥ 3 or 4 in the total of pig days. ^a–c^Means without a common superscript are different (*P* < 0.05) in frequency of diarrhea ≥ 3. ^A–C^Means without a common superscript are different (*P* < 0.05) in frequency of diarrhea ≥ 4

Pigs in the ZNO group had the lowest (*P* < 0.05) fecal score from d 1 to 10 PI among dietary treatments (Fig. 1). The incidence of diarrhea was 32.09% in control, 30.41% in monoglycerides, 4.01% in ZNO, and 22.53% in antibiotic, while the severity of diarrhea was 19.26% in control, 16.22% in monoglycerides, 0.31% in ZNO, and 12.35% in antibiotic, respectively (Fig. 2). The incidence of diarrhea (fecal score ≥ 3) was lower (*P* < 0.05) in ZNO and antibiotic groups than control and monoglycerides groups. The severity of diarrhea (fecal score ≥ 4) in ZNO and antibiotic groups was also lower than that in control, but there was no difference observed in the severity of diarrhea between monoglycerides and antibiotic groups. The ZNO group had the lowest incidence and severity of diarrhea throughout the experimental period.

**Fig. 3:**
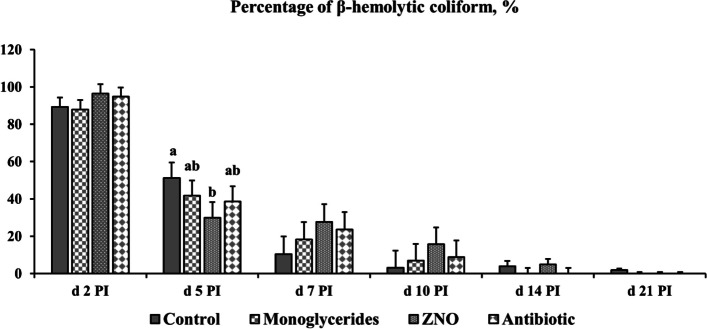
The percentage (%) of β-hemolytic coliforms in fecal samples of enterotoxigenic *Escherichia coli* F18-challenged pigs fed diets supplemented with monoglycerides, high-dose zinc oxide (ZNO), or antibiotic. No β-hemolytic coliforms were observed in the fecal samples of pigs before *Escherichia coli* challenge. β-Hemolytic coliforms were only observed in control pigs on d 21 post-inoculation (PI). Each least squares mean represents 14–15 observations on d 2 and 5 PI and each least squares mean represents 8–9 observations on d 7, 10, 14, and 21 PI. ^a,b^Means without a common superscript are different (*P* < 0.05)

No β-hemolytic coliforms were identified in fecal samples of pigs in all groups prior to ETEC inoculation. Βeta-hemolytic coliforms were identified in all pigs’ feces on d 2 PI. Pigs in ZNO group had lower (*P* < 0.05) percentage of β-hemolytic coliforms in feces on d 5 PI than pigs in control, while no difference was observed among monoglycerides, ZNO, and antibiotic groups (Fig. 3). No difference was observed in the percentage of β-hemolytic coliforms in feces among all dietary treatments on d 7, 10, 14, and 21 PI.

### Systemic immunity

No difference was observed in serum TNF-α concentrations among all treatments at d 0 before ETEC inoculation, and at d 2, 5, and 21 PI (Table 3). Dietary supplements tended (*P* = 0.07) to impact serum TNF-α on d 14 PI, pigs fed with ZNO had the lowest TNF-α and pigs fed with control had the highest level of TNF-α among all treatments. Pigs in monoglycerides group had lower (*P* < 0.05) serum CRP than pigs in the antibiotic group on d 0 before ETEC inoculation. Supplementation of ZNO reduced (*P* < 0.10 and *P* < 0.05) serum CRP on d 14 and 21 PI, tended (*P* = 0.06) to reduce serum haptoglobin on d 0, and reduced (*P* < 0.05) serum haptoglobin on d 2 and 5 PI. Pigs fed with monoglycerides also had lower (*P* < 0.05) serum haptoglobin on d 5 PI, compared with control pigs.

**Table 3:** Serum tumor necrosis factor-alpha and acute-phase proteins in enterotoxigenic *Escherichia coli* F18-challenged weaned pigs fed experimental diets

Item^d^	Control	Monoglycerides	ZNO^e^	Antibiotic	SEM	*P*-value
d 0 before inoculation						
TNF-α, pg/mL	187.51	197.13	208.46	226.26	38.15	0.90
CRP, µg/mL	7.57^ab^	5.56^b^	8.26^ab^	11.88^a^	2.61	< 0.05
Haptoglobin, µg/mL	1,531.22^ab^	1,236.94^ab^	756.45^b^	2,217.13^a^	402.98	0.06
d 2 post-inoculation						
TNF-α, pg/mL	236.35	277.77	235.19	232.65	42.62	0.86
CRP, µg/mL	9.15	5.95	8.96	10.59	2.06	0.18
Haptoglobin, µg/mL	1,637.06^a^	1,783.59^a^	818.70^b^	1,724.84^a^	284.28	< 0.05
d 5 post-inoculation						
TNF-α, pg/mL	271.96	334.52	291.19	271.28	51.42	0.81
CRP, µg/mL	6.49	6.98	4.19	8.76	1.62	0.21
Haptoglobin, µg/mL	1,800.29^a^	1,118.21^b^	365.53^c^	1,300.01^ab^	224.95	< 0.01
d 14 post-inoculation^f^						
TNF-α, pg/mL	462.00^a^	254.71^ab^	213.75^b^	400.18^ab^	69.97	0.07
CRP, µg/mL	7.95^ab^	8.42^a^	6.92^b^	8.21^ab^	1.56	0.09
Haptoglobin, µg/mL	1,280.88	1,250.54	551.28	1,002.63	332.56	0.42
d 21 post-inoculation^f^						
TNF-α, pg/mL	421.64	275.89	273.94	290.19	79.20	0.49
CRP, µg/mL	9.10^a^	9.12^a^	4.18^b^	8.23^a^	1.33	< 0.01
Haptoglobin, µg/mL	626.76	770.43	174.50	130.36	201.65	0.11

^a–c^Means without a common superscript are different (*P* < 0.05)

^d^*TNF-α* Tumor necrosis factor-alpha, *CRP* C-reactive protein. Each least squares mean represents 14–15 observations

^e^*ZNO* High-dose zinc oxide

^f^Each least squares mean represents 8–9 observations

### Intestinal morphology

On d 5 PI, pigs in ZNO had more (*P* < 0.05) goblet cell numbers per villus, greater (*P* < 0.05) villus area and VH, and higher (*P* < 0.05) VH:CD in duodenum than pigs in other treatments (Table 4). Supplementation of monoglycerides, ZNO, or antibiotic reduced (*P* < 0.05) ileal CD compared with control. Consistently, pigs in ZNO group tended (*P* = 0.06) to have the biggest VH:CD in the ileum, followed by pigs in monoglycerides and antibiotic groups. On d 21 PI, pigs supplemented with ZNO tended (*P* = 0.07) to have more goblet cells per villus, and had largest (*P* < 0.05) villus area and highest (*P* < 0.05) VH in the duodenum, when compared with other treatments.

**Table 4:** Intestinal morphology of enterotoxigenic *Escherichia coli* F18-challenged weaned pigs fed experimental diets

Item^c^	Control	Monoglycerides	ZNO^d^	Antibiotic	SEM	*P*-value
d 5 PI						
Duodenum						
Goblet cells, per villus	10.97^b^	8.61^b^	18.58^a^	12.86^ab^	2.40	< 0.05
Villus area, µm^2^	12,473^b^	14,342^b^	20,838^a^	16,097^b^	1549	< 0.01
Villus height, µm	183.56^b^	190.73^b^	274.42^a^	216.75^b^	13.11	< 0.01
Villus width, µm	70.92	81.33	81.22	79.10	4.62	0.13
Crypt depth, µm	245.58	236.32	237.64	251.08	21.00	0.88
Crypt width, µm	25.02	24.88	24.99	24.24	1.12	0.91
VH:CD	0.77^b^	0.83^b^	1.21^a^	0.91^b^	0.072	< 0.01
Jejunum						
Goblet cells, per villus	4.87	6.95	4.73	4.08	1.53	0.60
Villus area, µm^2^	12,434	12,002	16,387	13,376	1817	0.30
Villus height, µm	200.14	205.14	236.62	213.79	17.42	0.42
Villus width, µm	64.45	61.02	69.64	63.84	3.86	0.28
Crypt depth, µm	147.26	135.38	138.27	145.15	13.03	0.81
Crypt width, µm	22.73	23.42	24.23	25.18	1.049	0.23
VH:CD	1.41	1.68	1.79	1.56	0.16	0.35
Ileum						
Goblet cells, per villus	15.38	16.40	17.00	17.28	2.90	0.97
Villus area, µm^2^	12,826	9,723	11,251	11,128	1101	0.11
Villus height, µm	184.86	174.00	193.78	173.11	12.18	0.58
Villus width, µm	67.95	64.39	60.84	63.62	2.92	0.27
Crypt depth, µm	170.20^a^	123.54^b^	136.34^b^	137.90^b^	12.29	< 0.05
Crypt width, µm	22.34	22.85	23.64	24.13	0.64	0.50
VH:CD	1.20^b^	1.36^ab^	1.57^a^	1.34^ab^	0.110	0.06
d 21 PI^e^						
Duodenum						
Goblet cells, per villus	28.80^ab^	25.04^ab^	37.07^a^	21.44^b^	3.98	0.07
Villus area, µm^2^	30,642^ab^	24,823^b^	34,452^a^	25,941^b^	2336	< 0.05
Villus height, µm	309.02^b^	278.81^b^	366.04^a^	271.89^b^	18.64	< 0.05
Villus width, µm	98.56	88.51	96.46	97.05	3.17	0.18
Crypt depth, µm	275.04	272.96	282.99	250.34	14.52	0.41
Crypt width, µm	27.29	28.85	27.28	27.05	0.90	0.52
VH:CD	1.21	1.15	1.37	1.18	0.101	0.42
Jejunum						
Goblet cells/villus	8.89	10.08	11.57	9.08	1.41	0.45
Villus area, µm^2^	18,156	18,460	18,590	19,026	1343	0.97
Villus height, µm	257.12	276.95	282.69	279.23	15.19	0.66
Villus width, µm	73.33	69.45	71.08	73.11	2.71	0.71
Crypt depth, µm	165.89	170.21	180.80	165.23	11.99	0.56
Crypt width, µm	26.48	26.67	26.13	26.44	0.80	0.97
VH:CD	1.69	1.70	1.71	1.79	0.15	0.95
Ileum						
Goblet cells, per villus	18.40	19.74	22.26	18.18	3.45	0.73
Villus area, µm^2^	14,508	15,979	16,654	15,200	2130	0.57
Villus height, µm	220.74	239.54	248.57	236.31	17.99	0.42
Villus width, µm	70.96	75.91	73.84	71.49	5.13	0.66
Crypt depth, µm	166.96	154.41	161.84	148.58	14.19	0.59
Crypt width, µm	27.50	28.50	25.85	26.24	1.038	0.21
VH:CD	1.40	1.70	1.90	1.70	0.12	0.22

^a,b^Means without a common superscript are different (*P* < 0.05)

^c^*PI* Post-inoculation, *VH:CD* Villus height-to-crypt depth ratio. Each least squares mean represents 6 observations

^d^*ZNO* High-dose zinc oxide

^e^Each least squares mean represents 8–9 observations

### Immunohistochemistry

Supplementation of ZNO or antibiotic reduced (*P* < 0.05) neutrophil counts in ileal villi on d 5 PI compared with control (Table 5). However, no significant differences in neutrophil counts were observed among monoglycerides, ZNO, and antibiotic groups. Pigs supplemented with ZNO had the lowest (*P* < 0.05) number of macrophages in ileal villi among all treatments on d 5 PI. Pigs fed with antibiotic also had significantly lower (*P* < 0.05) recruitment of macrophages in ileal villi than control group, but comparable to that in pigs fed with monoglycerides.

**Table 5:** Number of neutrophils and macrophages in the ileum of enterotoxigenic *Escherichia coli* F18-challenged weaned pigs fed experimental diets

Item	Control	Monoglycerides	ZNO^d^	Antibiotic	SEM	*P*-value
d 5 post-inoculation^e^						
Neutrophils	2,596^a^	1,759^ab^	1,382^b^	1,406^b^	382	< 0.05
Macrophages	2,236^a^	1,715^ab^	676^c^	1,085^bc^	369	< 0.05

^a–c^Means without a common superscript are different (*P* < 0.05)

^d^*ZNO* High-dose zinc oxide

^e^Each least squares mean represents 6 observations

### Intestinal barrier and innate immunity

**Fig. 4:**
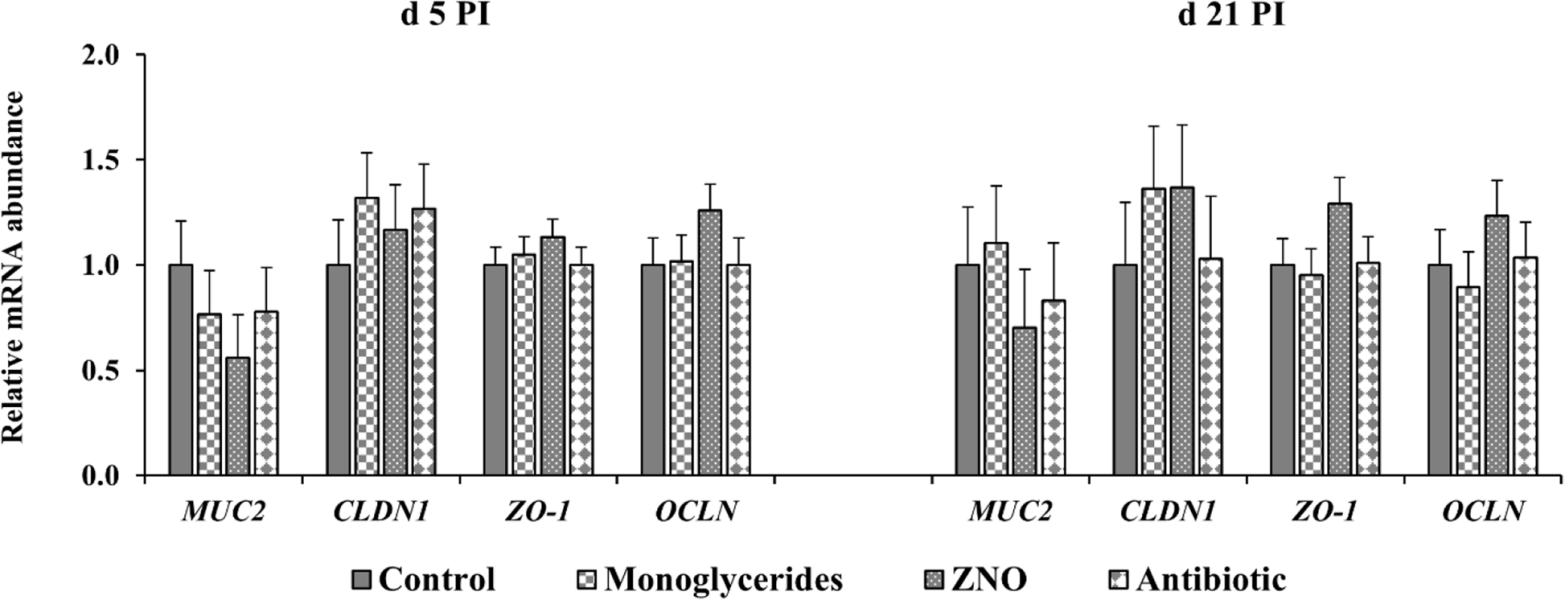
Relative mRNA abundance of genes in jejunal mucosa of enterotoxigenic *Escherichia coli* F18-challenged weaned pigs fed diets supplemented with monoglycerides, high-dose zinc oxide, or antibiotic. Each least squares mean represents 6–9 observations. PI, Post-inoculation; *MUC2*, Mucin 2; *CLDN1*, Claudin-1; *ZO-1*, Zonula occludens-1; *OCLN*, Occludin

**Fig. 5:**
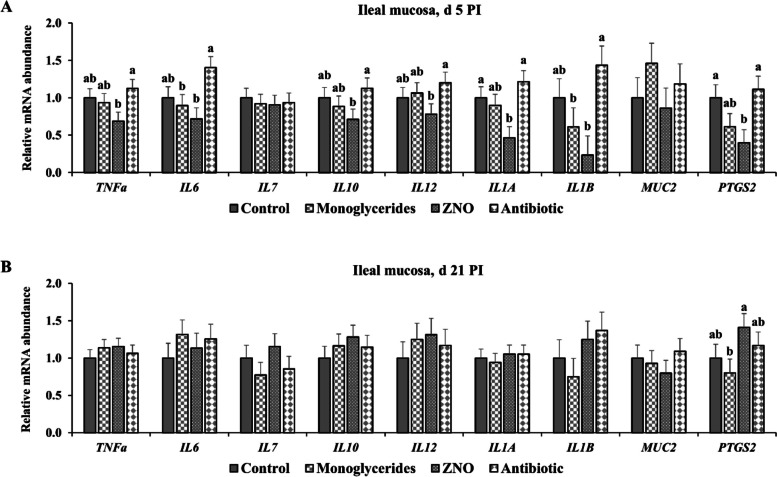
Relative mRNA abundance of genes in ileal mucosa of enterotoxigenic *Escherichia coli* F18-challenged pigs supplemented with monoglycerides, high-dose zinc oxide, or antibiotic on d 5 (**A**) and 21 PI (**B**). ^a,b^Means without a common superscript are different (*P* < 0.05). Each least squares mean represents 6–9 observations. PI, Post-inoculation; *TNFa*, Tumor necrosis factor-alpha; *IL6*, Interleukin 6; *IL7*, Interleukin 7; *IL10*, Interleukin 10; *IL12*, Interleukin 12; *IL1A*, Interleukin-1 alpha, *IL1B*, Interleukin-1 beta; *MUC2*, Mucin 2, and *PTGS2*, Prostaglandin-endoperoxide synthase 2

No differences were observed in the mRNA expression of *MUC2*, *CLDN1*, *ZO-1*, and *OCLN* in jejunal mucosa of weaned pigs among different treatments on d 5 and 21 PI (Fig. 4). On d 5 PI, pigs fed with ZNO had lower (*P* < 0.05) mRNA expression of *TNFa*, *IL6*, *IL10*, *IL12*, *IL1A*, *IL1B*, and *PTGS2* in ileal mucosa, compared with other treatments (Fig. 5). However, no difference in the expression of listed genes was observed between pigs supplemented with monoglycerides or ZNO. Pigs supplemented with monoglycerides expressed lowest (*P* < 0.05) *PTGS2* in ileal mucosa compared with other treatments on d 21 PI.

### Metabolite profiles in serum

A total of 483 (165 identified and 318 unidentified) metabolites were detected in serum samples. Based on statistical threshold and VIP scores, pantothenic acid and fructose were up-regulated by ZNO, compared with the pigs in control group on d 5 PI (Table 6). Supplementation of monoglycerides changed the relative abundances of 14 metabolites (7 up-regulated and 7 down-regulated) compared with ZNO, and upregulated lactose and cellobiose compared with antibiotics on d 5 PI. On d 14 PI, supplementation of ZNO changed abundances of 10 metabolites (7 up-regulated and 3 down-regulated) compared with control. Supplementation of monoglycerides up-regulated 2 metabolites (hippuric acid and indole-3-propionic acid) and down-regulated 8 metabolites (including glutaric acid, serotonin, mannose, etc.) compared with pigs in the ZNO. Pigs fed with antibiotics had greater abundance of hippuric acid and indole-3-propionic acid, but had lower thymine, pantothenic acid, glycerol, and piperidone compared with the pigs in the ZNO group. Limited differential metabolites were identified when comparing control vs. monoglycerides, and control vs. antibiotic throughout the experiment (data not shown).

**Table 6:** Serum metabolites that differed among the dietary treatment groups

Metabolite	Fold change^a^	VIP^b^	FDR^c^
Control vs. ZNO^d^, d 5 post-inoculation			
Pantothenic acid	0.32	1.97	0.156
Fructose	0.43	2.08	0.137
Monoglycerides vs. ZNO, d 5 post-inoculation			
Piperidone	0.12	2.05	0.093
Kynurenine	0.34	1.87	0.099
β-Alanine	0.36	1.78	0.099
Xylonic acid	0.41	1.82	0.099
Nicotinamide	0.44	1.83	0.099
Pantothenic acid	0.46	1.77	0.099
Glutaric acid	0.48	1.60	0.141
Hippuric acid	2.08	1.56	0.157
6-Oxopiperidine-2-carboxylic acid	2.28	1.75	0.099
Histidine	2.29	1.50	0.177
Sucrose	2.43	1.72	0.110
Indoxyl sulfate	2.45	1.59	0.141
Glycyl tyrosine	2.69	1.59	0.141
α-Aminoadipic acid	4.48	1.51	0.177
Monoglycerides vs. Antibiotic, d 5 post-inoculation			
Lactose	2.38	2.49	0.034
Cellobiose	2.41	2.52	0.034
Control vs. ZNO, d 14 post-inoculation			
Piperidone	0.09	1.79	0.014
Glutaric acid	0.30	1.21	0.146
Glycerol-3-galactoside	0.33	1.47	0.067
Oleic acid	0.37	1.25	0.129
5-Methoxytryptamine	0.38	1.47	0.067
Mannose	0.42	1.81	0.014
Thymine	0.43	1.71	0.024
Conduritol-β-epoxide	2.18	1.62	0.037
Hippuric acid	3.36	1.51	0.062
Indole-3-propionic acid	3.48	1.48	0.067
Monoglycerides vs. ZNO, d 14 post-inoculation			
Piperidone	0.10	1.70	0.031
Glycerol	0.22	1.75	0.031
Taurine	0.22	1.14	0.194
Glutaric acid	0.25	1.35	0.118
Serotonin	0.30	1.62	0.032
Oleic acid	0.37	1.28	0.137
Glycerol-3-galactoside	0.40	1.31	0.129
Mannose	0.47	1.75	0.031
Hippuric acid	3.31	1.43	0.078
Indole-3-propionic acid	5.25	1.63	0.032
ZNO vs. Antibiotic, d 14 post-inoculation			
Hippuric acid	0.34	1.72	0.158
Indole-3-propionic acid	0.43	1.70	0.158
Thymine	2.09	2.05	0.070
Pantothenic acid	2.60	1.70	0.158
Glycerol	2.73	1.79	0.150
Piperidone	4.84	1.83	0.150

^a^Fold change values less than one indicate that the differential metabolites were reduced in the Control compared to ZNO or Monoglycerides compared to ZNO or Monoglycerides compared to Antibiotic or ZNO compared to Antibiotic, respectively

^b^*VIP* Variable importance in projection

^c^*FDR* False discovery rate

^d^*ZNO* High-dose zinc oxide

**Fig. 6:**
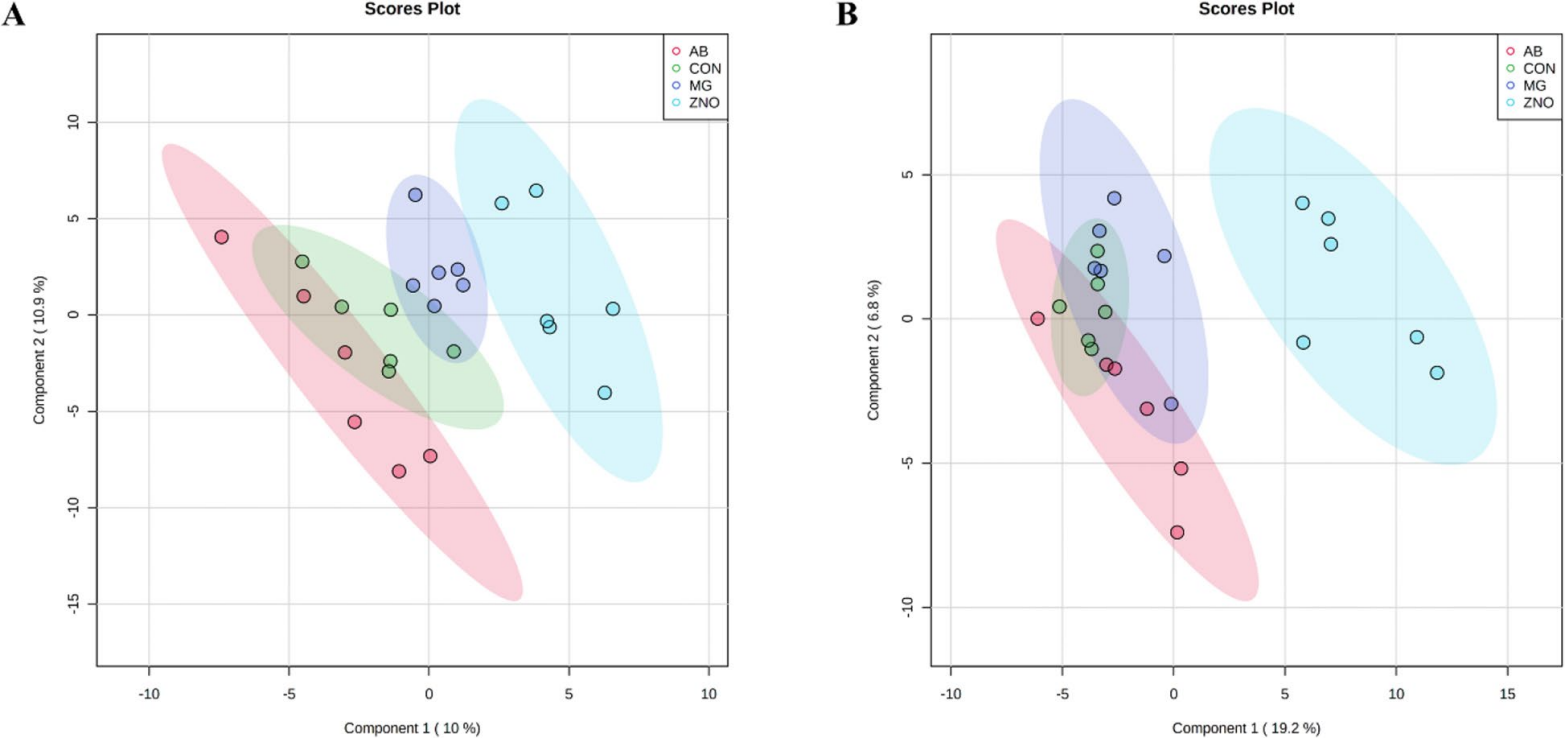
Partial Least Squares Discriminant Analysis (PLS-DA) 2D score plot of the metabolites in serum showed separated clusters between the CON and ZNO, MG and ZNO, MG and AB, and ZNO and AB groups on d 5 (**A**) and/or d 14 (**B**) post-inoculation, respectively. CON = Control; MG = Monoglycerides; ZNO = High-dose zinc oxide; AB = Antibiotic. Shaded areas in different colors represent in 95% confidence interval

Based on the identified metabolites and VIP scores, a PLS-DA score with 95% confidence ranges (shaded areas) showed a clear separation between control and ZNO, between monoglycerides and ZNO, between monoglycerides and antibiotic, and between ZNO and antibiotic groups on d 5 PI (Fig. 6A) and/or d 14 PI (Fig. 6B). To further explore the metabolic profile differences among dietary treatments, PLS-DA was performed for the following comparisons: (1) control vs. ZNO, (2) monoglycerides vs. ZNO, (3) monoglycerides vs. antibiotic, and (4) ZNO vs. antibiotic on d 5 and 14 PI. The score plot again distinguished control from ZNO (Fig. S1A and B), monoglycerides from ZNO (Fig. S1C and D), monoglycerides from antibiotic (Fig. S2A and B), and ZNO from antibiotic (Fig. S2C and D).

Pathway analysis and metabolite set enrichment analysis were performed on the identified metabolites in serum with VIP > 1 (Table 7). On d 5 PI, taurine and hypotaurine metabolism and phenylalanine metabolism were the most affected metabolic pathways in a comparison of control vs. monoglycerides (Fig. S3A and B). Arginine biosynthesis, β-alanine metabolism, arginine and proline metabolism, pyruvate metabolism, citrate cycle (TCA cycle), glyoxylate and dicarboxylate metabolism, and glycolysis/gluconeogenesis were the most affected metabolic pathways when comparing control with ZNO (Fig. S4A and B). Citrate cycle, taurine and hypotaurine metabolism, and β-alanine metabolism were the most affected metabolic pathways when monoglyceride blend was compared with ZNO (Fig. S5A and B). Taurine and hypotaurine metabolism, nicotinate and nicotinamide metabolism, and β-alanine metabolism were the most affected metabolic pathways in a comparison of monoglycerides vs. antibiotic (Fig. S6A and B). β-Alanine metabolism and citrate cycle were the most affected metabolic pathways when comparing ZNO with antibiotic (Fig. S7A and B). On d 14 PI, glyoxylate and dicarboxylate metabolism and taurine and hypotaurine metabolism were the most affected metabolic pathways in a comparison of control vs. monoglycerides (Fig. S3C and D). Alanine, aspartate and glutamate metabolism, citrate cycle, glyoxylate and dicarboxylate metabolism, and pyrimidine metabolism were the most affected metabolic pathways when comparing control with ZNO (Fig. S4C and D). Citrate cycle, glyoxylate and dicarboxylate metabolism, alanine, aspartate and glutamate metabolism, and pyrimidine metabolism were the most affected metabolic pathways when monoglyceride blend was compared with ZNO (Fig. S5C and D), while citrate cycle was the most affected metabolic pathway in comparison of monoglycerides vs. antibiotic (Fig. S6C and D). Alanine, aspartate and glutamate metabolism, glyoxylate and dicarboxylate metabolism, citrate cycle, D-glutamine and D-glutamate metabolism, pyrimidine metabolism, arginine biosynthesis, and β-alanine metabolism were the most affected metabolic pathways when comparing ZNO with antibiotic (Fig. S7C and D).

**Table 7:** Significant impact pathways in serum that affected by the dietary treatment groups

Pathway name	Impact^a^	*P*-value^b^
Control vs. Monoglycerides, d 5 post-inoculation		
Taurine and hypotaurine metabolism	0.71	0.017
Phenylalanine metabolism	0.36	0.027
Control vs. ZNO^c^, d 5 post-inoculation		
Arginine biosynthesis	0.41	< 0.001
β-Alanine metabolism	0.40	0.026
Arginine and proline metabolism	0.34	0.006
Pyruvate metabolism	0.21	0.030
Citrate cycle (TCA cycle)	0.13	0.023
Glyoxylate and dicarboxylate metabolism	0.10	0.017
Glycolysis/Gluconeogenesis	0.10	0.046
Control vs. Antibiotic, d 5 post-inoculation		
Citrate cycle (TCA cycle)	0.21	0.002
Glyoxylate and dicarboxylate metabolism	0.11	0.012
Phenylalanine metabolism	0.36	0.032
Monoglycerides vs. ZNO, d 5 post-inoculation		
Citrate cycle (TCA cycle)	0.13	0.024
Taurine and hypotaurine metabolism	0.71	0.025
β-Alanine metabolism	0.40	0.028
Monoglycerides vs. Antibiotic, d 5 post-inoculation		
Taurine and hypotaurine metabolism	0.71	0.001
Nicotinate and nicotinamide metabolism	0.19	0.010
β-Alanine metabolism	0.40	0.026
ZNO vs. Antibiotic, d5 post-inoculation		
β-Alanine metabolism	0.40	0.004
Citrate cycle (TCA cycle)	0.19	0.023
Control vs. Monoglycerides, d 14 post-inoculation		
Glyoxylate and dicarboxylate metabolism	0.21	0.014
Taurine and hypotaurine metabolism	0.29	0.022
Control vs. ZNO, d 14 post-inoculation		
Alanine, aspartate and glutamate metabolism	0.16	< 0.001
Citrate cycle (TCA cycle)	0.31	< 0.001
Glyoxylate and dicarboxylate metabolism	0.24	< 0.001
Pyrimidine metabolism	0.12	0.003
Control vs. Antibiotic, d 14 post-inoculation		
Citrate cycle (TCA cycle)	0.21	0.003
Glyoxylate and dicarboxylate metabolism	0.11	0.015
Taurine and hypotaurine metabolism	0.71	0.023
Monoglycerides vs. ZNO, d 14 post-inoculation		
Citrate cycle (TCA cycle)	0.28	< 0.001
Glyoxylate and dicarboxylate metabolism	0.24	< 0.001
Alanine, aspartate and glutamate metabolism	0.16	0.003
Pyrimidine metabolism	0.10	0.012
Monoglycerides vs. Antibiotic, d 14 post-inoculation		
Citrate cycle (TCA cycle)	0.19	0.017
ZNO vs. Antibiotic, d14 post-inoculation		
Alanine, aspartate and glutamate metabolism	0.36	< 0.001
Glyoxylate and dicarboxylate metabolism	0.22	< 0.001
Citrate cycle (TCA cycle)	0.27	< 0.001
D-Glutamine and D-glutamate metabolism	0.50	< 0.001
Pyrimidine metabolism	0.18	0.001
Arginine biosynthesis	0.12	0.010
β-Alanine metabolism	0.40	0.031

^a^Pathway impact value; cumulative percentage from the matched metabolite nodes that calculated from pathway topology analysis

^b^Original *P*-value calculated from the enrichment analysis

^c^*ZNO* High-dose zinc oxide

## Discussion

The present study investigated the potential of a monoglyceride blend containing butyric, caprylic, and capric acids in mitigating the adverse effects of ETEC F18 infection on systemic and intestinal immune responses, as well as intestinal health in weaning pigs. Additionally, the study identified metabolic changes resulting from monoglycerides supplementation, shedding light on potential mechanisms underlying the observed physiological responses.

Post-weaning diarrhea, a prevalent gastrointestinal disease occurring shortly after weaning, is often attributed to the adhesion and proliferation of ETEC F18 or F4 in the small intestine. Clinical signs typically include watery diarrhea, dirty appearance, stunted growth, dehydration, and lethargy [51, 59]. In this study, successful ETEC F18 infection was confirmed through fecal shedding of β-hemolytic coliforms and the manifestation of typical infection symptoms, including growth retardation and severe diarrhea. These observations are consistent with our previous research [50, 52]. The observed pattern of gradual recovery after the peak of infection (d 3 to 5 PI) also aligns with our previous studies using the same ETEC F18 strain [47, 52, 60]. The results of fecal score and the frequency of diarrhea indicated that supplementation of high-dose zinc oxide or antibiotics significantly reduces both the incidence and severity of diarrhea in weaned pigs infected with ETEC F18. However, the impact of dietary monoglycerides on diarrhea was limited.

ETEC toxins can disrupt the regulation of intestinal ion transporters, leading to fluid and electrolyte imbalances [61, 62]. Although the percentage of β-hemolytic coliforms in feces was similar across treatments post-infection, supplementation of high-dose zinc oxide notably reduced the β-hemolytic coliforms on d 5 PI, which may be attributed to zinc oxide’s antimicrobial properties and its ability to support intestinal barrier function and epithelial tissue regeneration [26, 28, 63]. Similarly, both monoglycerides and antibiotics showed comparable reductions in ETEC shedding, likely due to their antibacterial activity [37, 64]. This reduction corresponded with a decreased incidence of diarrhea across all supplemented groups.

It is well known that ETEC infection can disrupt essential intestinal functions, such as nutrient transport, epithelial barrier integrity, and immune function [13, 65]. All of these result in reduced digestive and absorptive capacity, and increased resource expenditure for maintaining intestinal homeostasis, ultimately leading to compromised performance in infected animals [51, 66, 67]. The beneficial effects of high-dose zinc oxide on intestinal morphology were significant, and supplementation with monoglycerides improved CD and VH:CD in the ileum of ETEC-infected pigs on d 5 PI, comparable to high-dose zinc oxide. However, there were limited changes in intestinal morphology on d 21 PI, likely due to the pigs’ recovery from ETEC infection. Consistent with our observations, previous studies have reported the positive effects of pharmacological doses of zinc oxide in managing post-weaning diarrhea caused by ETEC and have summarized its beneficial effects on growth performance, gastrointestinal tract health, and immunity [26]. Although the exact modes of action of carbadox are unclear, the observed changes in serum inflammatory markers and ileal morphology may be due to their ability to compete for sites important for nutrient absorption and ETEC colonization, thereby reducing resource costs and improving nutrient availability. Intestinal morphology results are also consistent with findings reported by Hung et al. [68], who observed that carbadox in the diet decreased CD and increased VH:CD in the small intestine of weaned pigs.

In addition to changes in intestinal morphology, high-dose zinc oxide and carbadox supplementation showed a mitigating effect on the recruitment of neutrophils and macrophages in the ileal villi. Supplementation with high-dose zinc oxide also reduced the relative gene expression of inflammatory cytokines (*TNFa*, *IL6*, *IL10*, *IL12*, *IL1A*, *IL1B*, and *PTGS2*) in ileal mucosa, indicating a moderating effect on the intestinal immune response. Although monoglycerides supplementation partially attenuated intestinal inflammation, its efficacy was not comparable to that of high-dose zinc oxide. The observed changes in the supplementation of monoglycerides suggest reduced intestinal epithelial cell renewal and attenuated inflammatory responses, indicating reduced energy and nutritional costs similar to conventional practices [68]. These findings also suggest that supplementing monoglycerides may overcome primary obstacles associated with the use of organic acids as feed additives, including undesirable losses in the upper intestine and unfavorable taste and aroma. The antibacterial effects of organic acids and their monoglycerides against *Escherichia coli* have been verified through numerous in vitro studies [30, 38, 41, 69]. The biological activity of butyric acid, which constitutes a major portion of our glyceride blend (~ 60%), has been well documented, including its modulation of various cellular responses via histone deacetylase inhibition and G-protein-coupled receptor activation in various cell types [36, 37, 70, 71], further supporting our findings.

Moreover, local inflammation can influence systemic immunity, and immune activation by external factors can exacerbate the performance status during the weaning period due to metabolic changes [72–74]. For instance, ETEC infection activates immune cells and increases the secretion of pro-inflammatory cytokines [47, 52, 75], leading to alterations in the absorption and utilization of nutrients or energy, including anorexia, decreased gut motility, and increased hepatic acute-phase protein synthesis [73, 76, 77]. Supplementation with high-dose zinc oxide was associated with a significant reduction in inflammatory biomarkers throughout the experiment, and an anti-inflammatory effect of monoglycerides was also observed during peak infection. This finding is supported by observations reported by Tian et al. [78], where inclusion of glycerol butyrate in pig diet reduced pro-inflammatory factors (*TNFa*, *IL6*, and *IL1B)* in jejunum and ileum to ETEC infection by inhibiting the NF-κB/MAPK pathway.

Given the biological effects of high-dose zinc oxide discussed earlier and the observed changes in diarrhea, intestinal morphology, and intestinal and serum inflammatory markers, it is not surprising that the pigs fed with high-dose zinc oxide had the greatest growth performance throughout the experimental period among all treatments. On the other hand, carbadox supplementation reduced feed intake compared to high-dose zinc oxide, but feed efficiency was higher than that of monoglycerides throughout the post-challenge period. These results reflect the multifactorial nature of animal growth and suggest that high-dose zinc oxide and antibiotics are likely to exert their beneficial effects through different mechanisms [68]. In the present study, the monoglyceride blend had limited effects on the growth performance of weaned pigs infected with ETEC F18. This finding aligns with other research showing that dietary supplementation of SCFA or MCFA monoglycerides did not affect the performance of weaned pigs [79–82]. Recent studies in poultry also confirmed that dietary supplementation of monoglyceride blend (butyric, caprylic, and capric acids) did not affect the growth performance of early growth stage in broilers infected with necrotic enteritis [43, 83]. In this study, supplementation of monoglyceride blend reduced gain:feed ratio of ETEC-infected pigs. However, it is noteworthy that this change was the result of increased feed intake. The observed improvement in feed intake in pigs fed with monoglycerides is further supported by the previously discussed anti-inflammatory effects of monoglycerides. Weaning stress is associated with reduced nutrient and energy intake, which may not recover even two weeks after weaning [84, 85]. Thus, the potential impacts of the monoglyceride blend on the feed intake of newly weaned pigs need to be further investigated in a performance trial with a larger number of animals.

The physiological changes caused by external factors, such as nutritional interventions or disease, can be comprehensively evaluated through a metabolomics analysis, providing valuable insights into the underlying mechanisms [86, 87]. In this study, pigs supplemented with high-dose zinc oxide exhibited significant alterations in serum metabolites primarily associated with carbohydrate and amino acid metabolism, compared to pigs in the control and monoglycerides groups. These changes are consistent with the mechanistic measurement results discussed earlier, and are also in line with the inferred effects suggested by other research related to nutrient and energy availability [68]. For example, the citrate cycle is a major metabolic pathway regulated to meet diverse cellular metabolic needs, including playing an important role in energy production and providing intermediates required for biosynthesis [88]. Recent studies have shown that these intermediates are also involved in cell signaling and have diverse functions, such as the regulation of chromatin modification and DNA methylation, as well as immunomodulation [86, 89].

Interestingly, monoglycerides supplementation had limited effects on serum metabolites compared to the control; however, significant pathway alterations were observed in serum metabolites when pigs were supplemented with monoglycerides. Specifically, taurine and hypotaurine metabolism was one of the metabolic pathways significantly affected by the supplementation of monoglycerides during the peak of ETEC infection. Taurine and hypotaurine are known to play crucial roles in cellular homeostasis and antioxidant responses [90, 91]. Similar to high-dose zinc oxide, carbadox supplementation had impacts on carbohydrate and amino acid metabolism in serum metabolites compared to control or monoglycerides. These changes include alterations in the citrate cycle and β-alanine metabolism. β-Alanine is a naturally occurring amino acid involved in the synthesis of carnosine, which exhibits beneficial biological activity, including antioxidant and anti-inflammatory properties [92–94]. Additionally, it has been reported that Mas-related G protein-coupled receptors, specifically responsive to β-alanine, may have beneficial effects on immune stress and homeostasis [95, 96].

## Conclusions

In conclusion, the findings of this study suggest that supplementation of monoglyceride blend including C4, C8, and C10 saturated fatty acids may enhance disease resistance by mitigating intestinal and systemic inflammation in weaned pigs challenged with enterotoxigenic *Escherichia coli* F18. Although the effects on performance and disease resistance were not comparable to that of high-dose zinc oxide, the efficacy was similar to the supplementation of carbadox. Additional research is needed to further evaluate the effects of monoglycerides supplementation on growth performance of weaned pigs under various external challenges in commercial conditions. Another area of research may be to explore combinations of monoglycerides with other acids, such as formic acid, as a potential alternative to conventional practices.

## Supplementary Information

**Additional file 1: Table S1** Gene-specific primer sequences and polymerase chain reaction conditions.

**Additional file 2:** **Fig. S1** Partial Least Squares Discriminant Analysis (PLS-DA) 2D score plot of the metabolites in serum showed separated clusters between the CON and ZNO (**A** and **B**), MG and ZNO (**C** and **D**) on d 5 (**A** and **C**) and d 14 (**B** and **D**) post-inoculation, respectively. CON, Control; MG, Monoglycerides; ZNO, High-dose zinc oxide. Shaded areas in different colors represent in 95% confidence interval. **Fig. S2** Partial Least Squares Discriminant Analysis (PLS-DA) 2D score plot of the metabolites in serum showed separated clusters between the MG and AB (**A** and **B**), ZNO and AB (**C** and **D**) on d 5 (**A** and **C**) and d 14 (**B** and **D**) post-inoculation, respectively. MG, Monoglycerides; ZNO, High-dose zinc oxide; AB, Antibiotic. Shaded areas in different colors represent in 95% confidence interval.

**Additional file 3:** **Fig. S3** Significantly changed pathways in serum between the control and monoglycerides groups on d 5 (**A**) and d 14 (**C**) post-inoculation, respectively. The *x*-axis represents the pathway impact values and the *y*-axis represents the −log(*P*) values from the pathway enrichment analysis. Metabolite set enrichment analysis shows the metabolic pathways were enriched in control compared with monoglycerides on d 5 (**B**) and d 14 (**D**) post-inoculation, respectively. Both pathway analysis and metabolite set enrichment analysis were performed using identified metabolites with VIP > 1. **Fig. S4** Significantly changed pathways in serum between the control and high-dose zinc oxide (ZNO) groups on d 5 (**A**) and d 14 (**C**) post-inoculation, respectively. The *x*-axis represents the pathway impact values and the *y*-axis represents the −log(*P*) values from the pathway enrichment analysis. Metabolite set enrichment analysis shows the metabolic pathways were enriched in control compared with ZNO on d 5 (**B**) and d 14 (**D**) post-inoculation, respectively. Both pathway analysis and metabolite set enrichment analysis were performed using identified metabolites with VIP > 1. **Fig. S5** Significantly changed pathways in serum between the monoglycerides and high-dose zinc oxide (ZNO) groups on d 5 (**A**) and d 14 (**C**) post-inoculation, respectively. The *x*-axis represents the pathway impact values and the *y*-axis represents the −log(P) values from the pathway enrichment analysis. Metabolite set enrichment analysis shows the metabolic pathways were enriched in monoglycerides compared with ZNO on d 5 (**B**) and d 14 (**D**) post-inoculation, respectively. Both pathway analysis and metabolite set enrichment analysis were performed using identified metabolites with VIP > 1. **Fig. S6** Significantly changed pathways in serum between the monoglycerides and antibiotic groups on d 5 (**A**) and d 14 (**C**) post-inoculation, respectively. The *x*-axis represents the pathway impact values and the *y*-axis represents the −log(*P*) values from the pathway enrichment analysis. Metabolite set enrichment analysis shows the metabolic pathways were enriched in monoglycerides compared with antibiotic on d 5 (**B**) and d 14 (**D**) post-inoculation, respectively. Both pathway analysis and metabolite set enrichment analysis were performed using identified metabolites with VIP > 1. **Fig. S7** Significantly changed pathways in serum between the high-dose zinc oxide (ZNO) and antibiotic groups on d 5 (**A**) and d 14 (**C**) post-inoculation, respectively. The *x*-axis represents the pathway impact values and the *y*-axis represents the −log(*P*) values from the pathway enrichment analysis. Metabolite set enrichment analysis shows the metabolic pathways were enriched in ZNO compared with antibiotic on d 5 (**B**) and d 14 (**D**) post-inoculation, respectively. Both pathway analysis and metabolite set enrichment analysis were performed using identified metabolites with VIP > 1.

**Additional file 4:** **Fig. S8** Intestinal morphology of enterotoxigenic *Escherichia coli* F18-challenged weaned pigs fed experimental diets on d 5 post-inoculation.

## Data Availability

All data generated or analyzed during this study are available from the corresponding author upon reasonable request.

## Abbreviations

ADFI: Average daily feed intake
ADG: Average daily gain
BW: Body weight
CD: Crypt depth
cDNA: Complementary DNA
CLDN1: Claudin-1
CRP: C-reactive protein
ETEC: Enterotoxigenic *Escherichia coli*
FDR: False discovery rate
IL6: Interleukin 6
IL7: Interleukin 7
IL10: Interleukin 10
IL12: Interleukin 12
IL1A: Interleukin-1 alpha
IL1B: Interleukin-1 beta
MCFA: Medium-chain fatty acids
MUC2: Mucin 2
OCLN: Occludin
PCR: Polymerase chain reaction
PI: Post-inoculation
PLS-DA: Partial least squares discriminant analysis
PTGS2: Prostaglandin-endoperoxide synthase 2
qRT-PCR: Quantitative real-time PCR
RNA: Ribonucleic acid
SCFA: Short-chain fatty acids
TNF-α/TNFa: Tumor necrosis factor-alpha
VH: Villus height
VIP: Variable importance in projection
ZNO: High-dose zinc oxide
ZO-1: Zonula occludens-1
