# Supplementary material for: Effects of monoglyceride blend on systemic and intestinal immune responses, and gut health of weaned pigs experimentally infected with a pathogenic Escherichia coli
Source: J Anim Sci Biotechnol. 2024 Oct 13;15:141. doi: 10.1186/s40104-024-01103-7 (PMC11479547; doi:10.1186/s40104-024-01103-7)
Supplement: Supplementary file 1 — Additional file 1: Table S1 Gene-specific primer sequences and polymerase chain reaction conditions. [file 40104_2024_1103_MOESM1_ESM.docx]

**Table S1** Gene-specific primer sequences and polymerase chain reaction conditions^1^

| **Gene^2^** | **Acc. No^3^** | **Forward primer (5´→3´)** | **Reverse primer (5´→3´)** |
| --- | --- | --- | --- |
| *MUC2* | AK231524 | CAACGGCCTCTCCTTCTCTGT | GCCACACTGGCCCTTTGT |
| *CLDN1* | NM_001244539 | TCTTAGTTGCCACAGCATGG | CCAGTGAAGAGAGCCTGACC |
| *ZO*-*1* | AJ318101 | CCGCCTCCTGAGTTTGATAG | CAGCTTTAGGCACTGTGCTG |
| *OCLN* | NM_001163647 | TTCATTGCTGCATTGGTGAT | ACCATCACACCCAGGATAGC  ACCATCACACCCAGGATAGC |
| *IL1A* | NM_214029 | CAGCCAACGGGAAGATTCTG | ATGGCTTCCAGGTCGTCAT |
| *IL1B* | NM_214055 | CCTTGAAACGTGCAATGATG | TTCAAGTCCCCTGTGAGGAG |
| *IL6* | NM_214399.1 | TAAGGGAAATGTCGAGGCCG | TTGTGTTCTTCATCCACTCGT |
| *IL7* | NM_214135 | CAACTGCACCAGCAAGGTTAAAG | AAGTCCCCCTGTCTTTTCTGTTC |
| *IL10* | NM_214041.1 | TCGGCCCAGTGAAGAGTTTC | GGAGTTCACGTGCTCCTTGA |
| *IL12* | NM_213993 | CGTGCCTCGGGCAATTATA | CGCAGGTGAGGTCGCTAGTT |
| *PTGS2* | AF207824 | ATAAGTGTGACTGCACCCGAAC | GGTGGGCTATCAATCAGATGTG |
| *TNFa* | NM_214022.1 | CGTGAAGCTGAAAGACAACCAG | GATGGTGTGAGTGAGGAAAAC |
| *18S* rRNA | NM_213940.1 | AGGAAAGCAGACATCGACCT | ACCTGGCTGTACTTCCCATC |

^1^Thermal cycling conditions were 95 °C for 20 s and 95 °C for 1 s, followed by 40 cycles with 20 s at 60 °C

^2^*MUC2* Mucin 2, *CLDN1* Claudin-1, *ZO*-*1* Zonula occludens-1, *OCLN* Occludin, *IL1A* Interleukin-1 alpha, *IL1B* Interleukin-1 beta, *IL6* Interleukin 6, *IL7* Interleukin 7, *IL10* Interleukin 10, *IL12* Interleukin 12, *PTGS2* Prostaglandin-endoperoxide synthase 2, *TNFa* Tumor necrosis factor-alpha, *18S* rRNA 18S ribosomal ribonucleic acid

^3^Accession number in GenBank database
