# Supplementary material for: Effects of monoglyceride blend on systemic and intestinal immune responses, and gut health of weaned pigs experimentally infected with a pathogenic Escherichia coli
Source: J Anim Sci Biotechnol. 2024 Oct 13;15:141. doi: 10.1186/s40104-024-01103-7 (PMC11479547; doi:10.1186/s40104-024-01103-7)
Supplement: Supplementary file 2 — Additional file 2: Fig. S1 Partial Least Squares Discriminant Analysis (PLS-DA) 2D score plot of the metabolites in serum showed separated clusters between the CON and ZNO (A and B), MG and ZNO (C and D) on d 5 (A and C) and d 14 (B and D) post-inoculation, respectively. CON, Control; MG, Monoglycerides; ZNO, High-dose zinc oxide. Shaded areas in different colors represent in 95% confidence interval. Fig. S2 Partial Least Squares Discriminant Analysis (PLS-DA) 2D score plot of the metabolites in serum showed separated clusters between the MG and AB (A and B), ZNO and AB (C and D) on d 5 (A and C) and d 14 (B and D) post-inoculation, respectively. MG, Monoglycerides; ZNO, High-dose zinc oxide; AB, Antibiotic. Shaded areas in different colors represent in 95% confidence interval. [file 40104_2024_1103_MOESM2_ESM.docx]

**
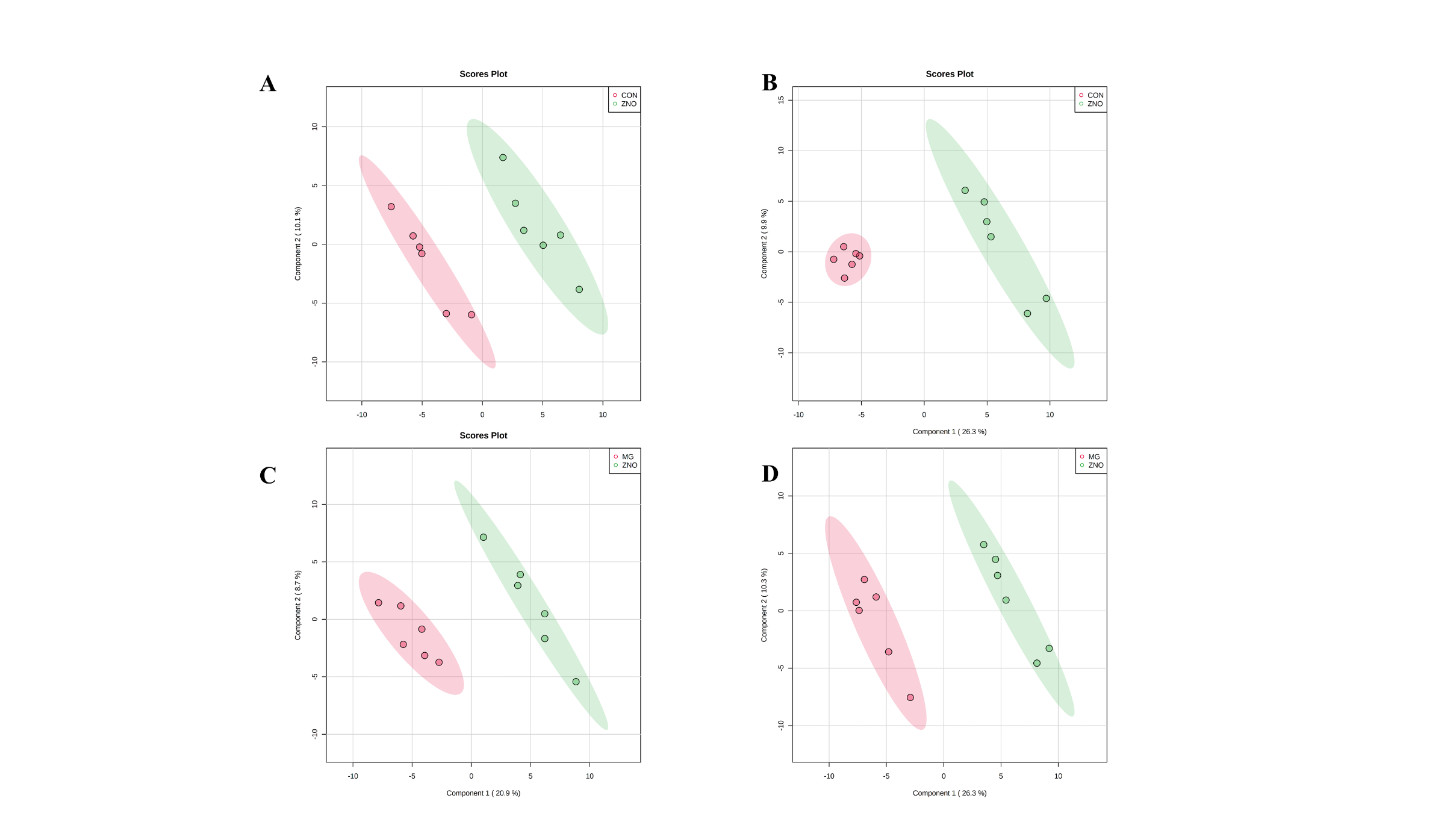
Fig. S1** Partial Least Squares Discriminant Analysis (PLS-DA) 2D score plot of the metabolites in serum showed separated clusters between the CON and ZNO (**A** and **B**), MG and ZNO (**C** and **D**) on d 5 (**A** and **C**) and d 14 (**B** and **D**) post-inoculation, respectively. CON, Control; MG, Monoglycerides; ZNO, High-dose zinc oxide. Shaded areas in different colors represent in 95% confidence interval

**
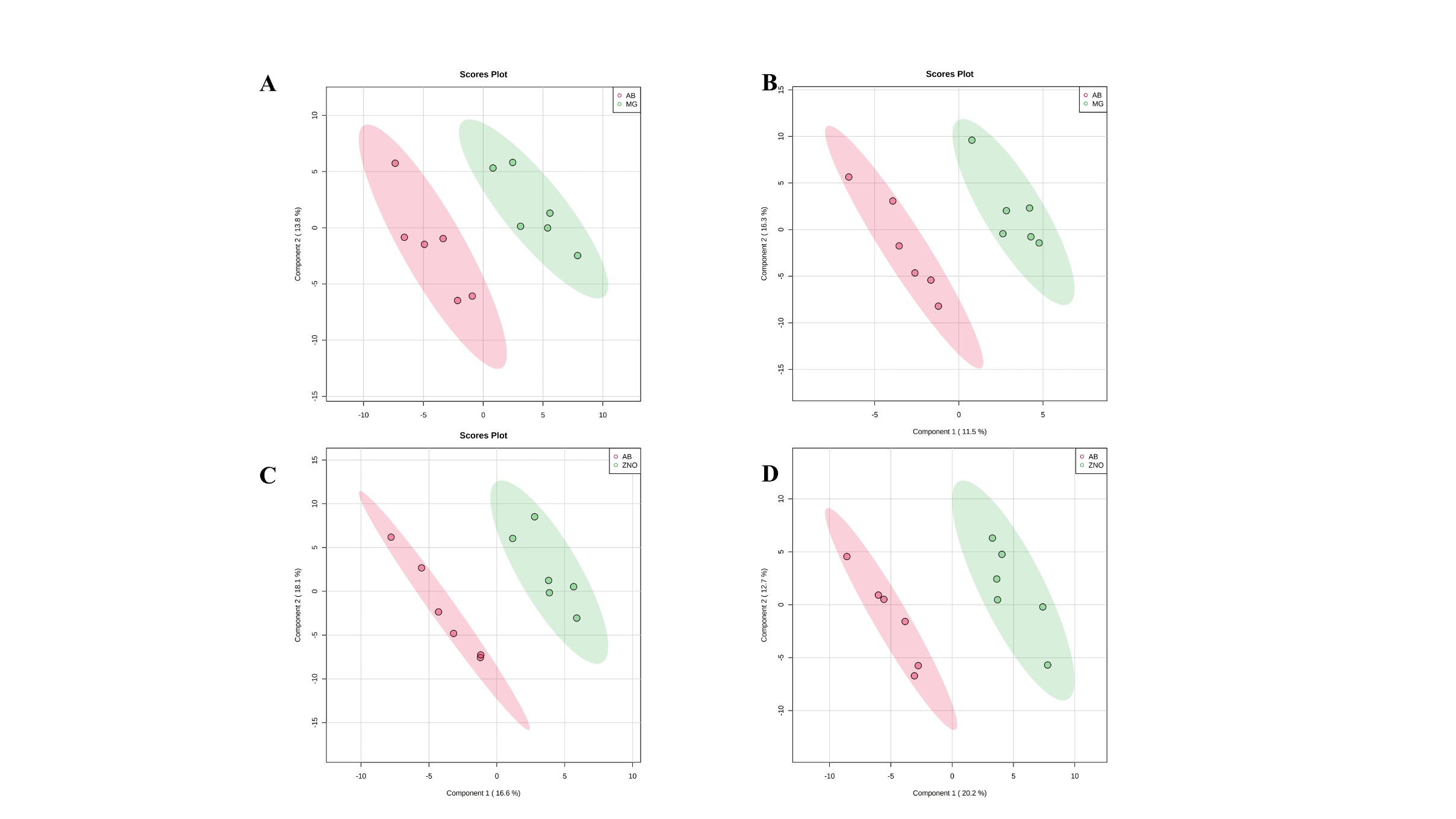
Fig. S2** Partial Least Squares Discriminant Analysis (PLS-DA) 2D score plot of the metabolites in serum showed separated clusters between the MG and AB (**A** and **B**), ZNO and AB (**C** and **D**) on d 5 (**A** and **C**) and d 14 (**B** and **D**) post-inoculation, respectively. MG, Monoglycerides; ZNO, High-dose zinc oxide; AB, Antibiotic. Shaded areas in different colors represent in 95% confidence interval
