# Supplementary material for: Effects of monoglyceride blend on systemic and intestinal immune responses, and gut health of weaned pigs experimentally infected with a pathogenic Escherichia coli
Source: J Anim Sci Biotechnol. 2024 Oct 13;15:141. doi: 10.1186/s40104-024-01103-7 (PMC11479547; doi:10.1186/s40104-024-01103-7)
Supplement: Supplementary file 3 — Additional file 3: Fig. S3 Significantly changed pathways in serum between the control and monoglycerides groups on d 5 (A) and d 14 (C) post-inoculation, respectively. The x-axis represents the pathway impact values and the y-axis represents the −log(P) values from the pathway enrichment analysis. Metabolite set enrichment analysis shows the metabolic pathways were enriched in control compared with monoglycerides on d 5 (B) and d 14 (D) post-inoculation, respectively. Both pathway analysis and metabolite set enrichment analysis were performed using identified metabolites with VIP > 1. Fig. S4 Significantly changed pathways in serum between the control and high-dose zinc oxide (ZNO) groups on d 5 (A) and d 14 (C) post-inoculation, respectively. The x-axis represents the pathway impact values and the y-axis represents the −log(P) values from the pathway enrichment analysis. Metabolite set enrichment analysis shows the metabolic pathways were enriched in control compared with ZNO on d 5 (B) and d 14 (D) post-inoculation, respectively. Both pathway analysis and metabolite set enrichment analysis were performed using identified metabolites with VIP > 1. Fig. S5 Significantly changed pathways in serum between the monoglycerides and high-dose zinc oxide (ZNO) groups on d 5 (A) and d 14 (C) post-inoculation, respectively. The x-axis represents the pathway impact values and the y-axis represents the −log(P) values from the pathway enrichment analysis. Metabolite set enrichment analysis shows the metabolic pathways were enriched in monoglycerides compared with ZNO on d 5 (B) and d 14 (D) post-inoculation, respectively. Both pathway analysis and metabolite set enrichment analysis were performed using identified metabolites with VIP > 1. Fig. S6 Significantly changed pathways in serum between the monoglycerides and antibiotic groups on d 5 (A) and d 14 (C) post-inoculation, respectively. The x-axis represents the pathway impact values and the y-axis represents the −log(P) valu [file 40104_2024_1103_MOESM3_ESM.docx]

**
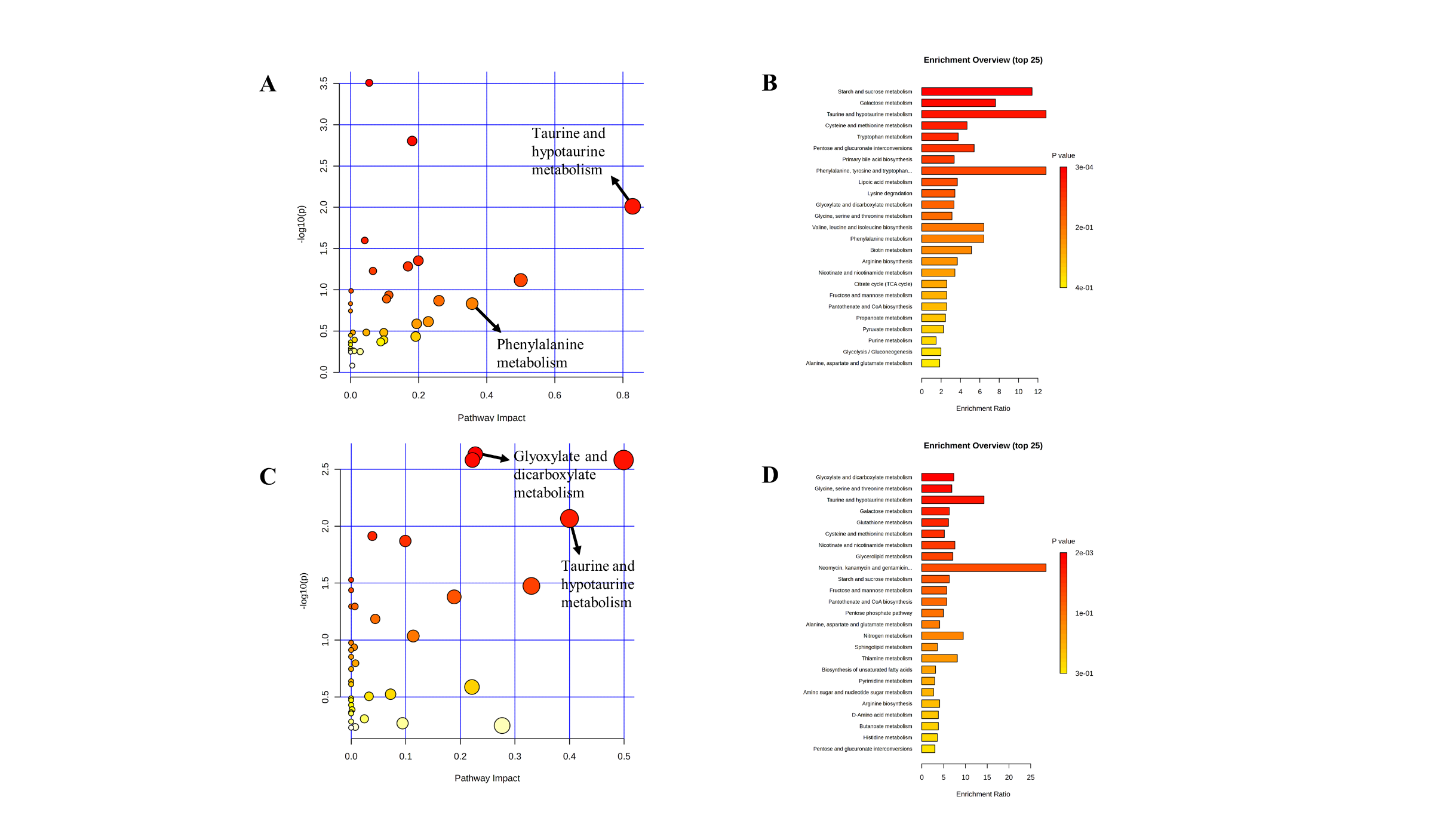
**

**Fig. S3** Significantly changed pathways in serum between the control and monoglycerides groups on d 5 (**A**) and d 14 (**C**) post-inoculation, respectively. The *x*-axis represents the pathway impact values and the *y*-axis represents the −log(*P*) values from the pathway enrichment analysis. Metabolite set enrichment analysis shows the metabolic pathways were enriched in control compared with monoglycerides on d 5 (**B**) and d 14 (**D**) post-inoculation, respectively. Both pathway analysis and metabolite set enrichment analysis were performed using identified metabolites with VIP > 1

**
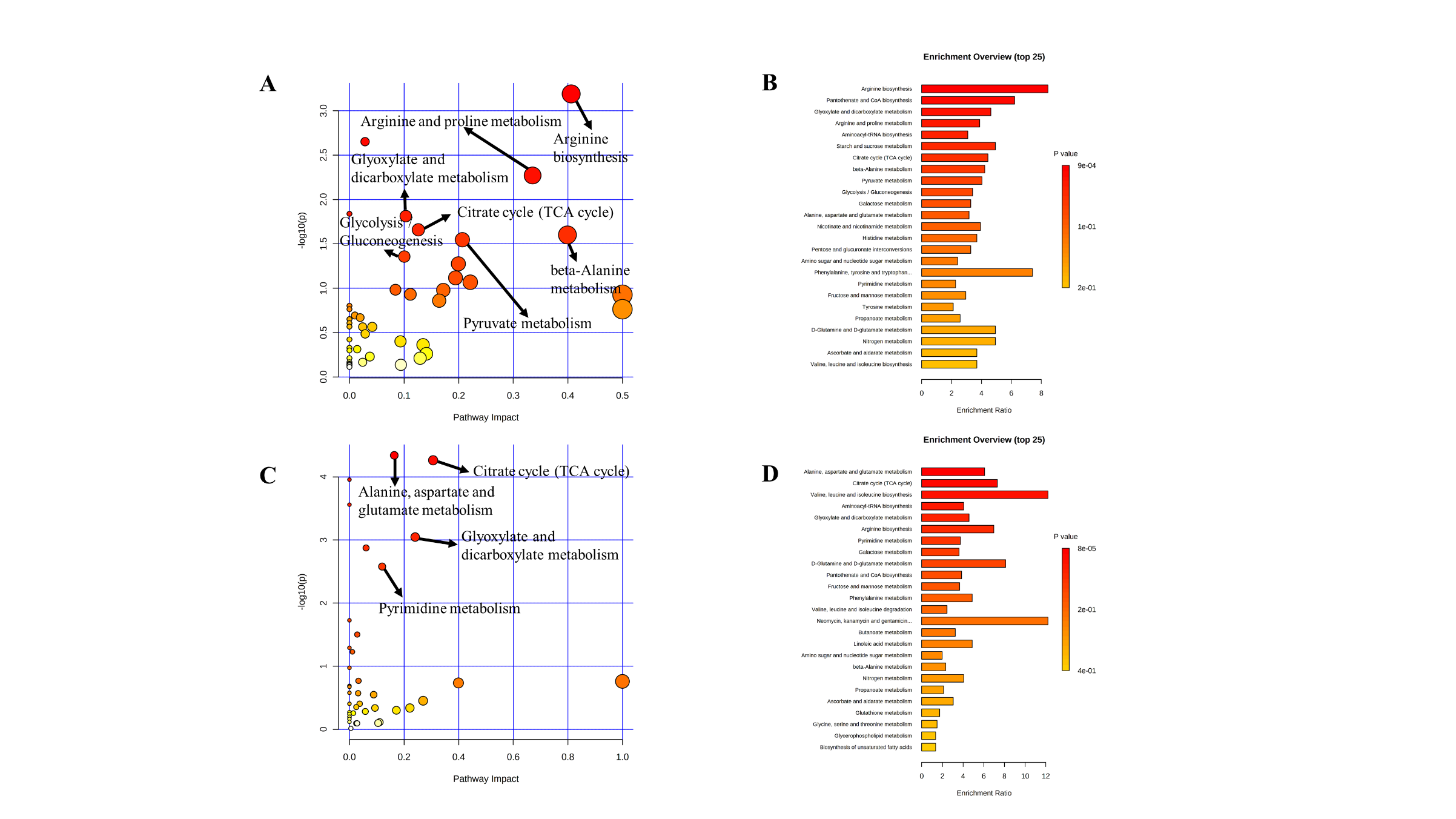
Fig. S4** Significantly changed pathways in serum between the control and high-dose zinc oxide (ZNO) groups on d 5 (**A**) and d 14 (**C**) post-inoculation, respectively. The *x*-axis represents the pathway impact values and the *y*-axis represents the −log(*P*) values from the pathway enrichment analysis. Metabolite set enrichment analysis shows the metabolic pathways were enriched in control compared with ZNO on d 5 (**B**) and d 14 (**D**) post-inoculation, respectively. Both pathway analysis and metabolite set enrichment analysis were performed using identified metabolites with VIP > 1


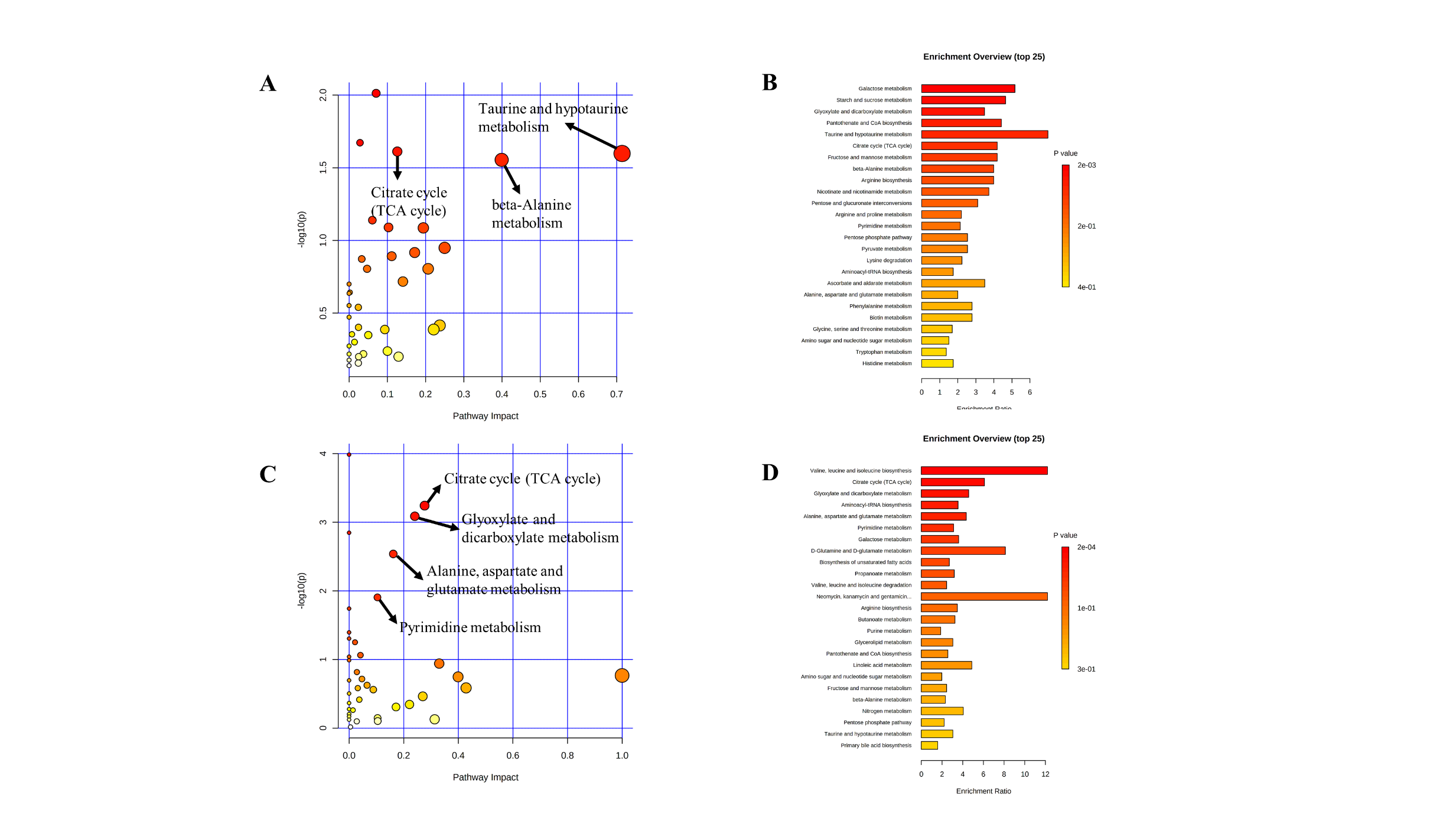
**Fig. S5** Significantly changed pathways in serum between the monoglycerides and high-dose zinc oxide (ZNO) groups on d 5 (**A**) and d 14 (**C**) post-inoculation, respectively. The *x*-axis represents the pathway impact values and the *y*-axis represents the −log(*P*) values from the pathway enrichment analysis. Metabolite set enrichment analysis shows the metabolic pathways were enriched in monoglycerides compared with ZNO on d 5 (**B**) and d 14 (**D**) post-inoculation, respectively. Both pathway analysis and metabolite set enrichment analysis were performed using identified metabolites with VIP > 1

**
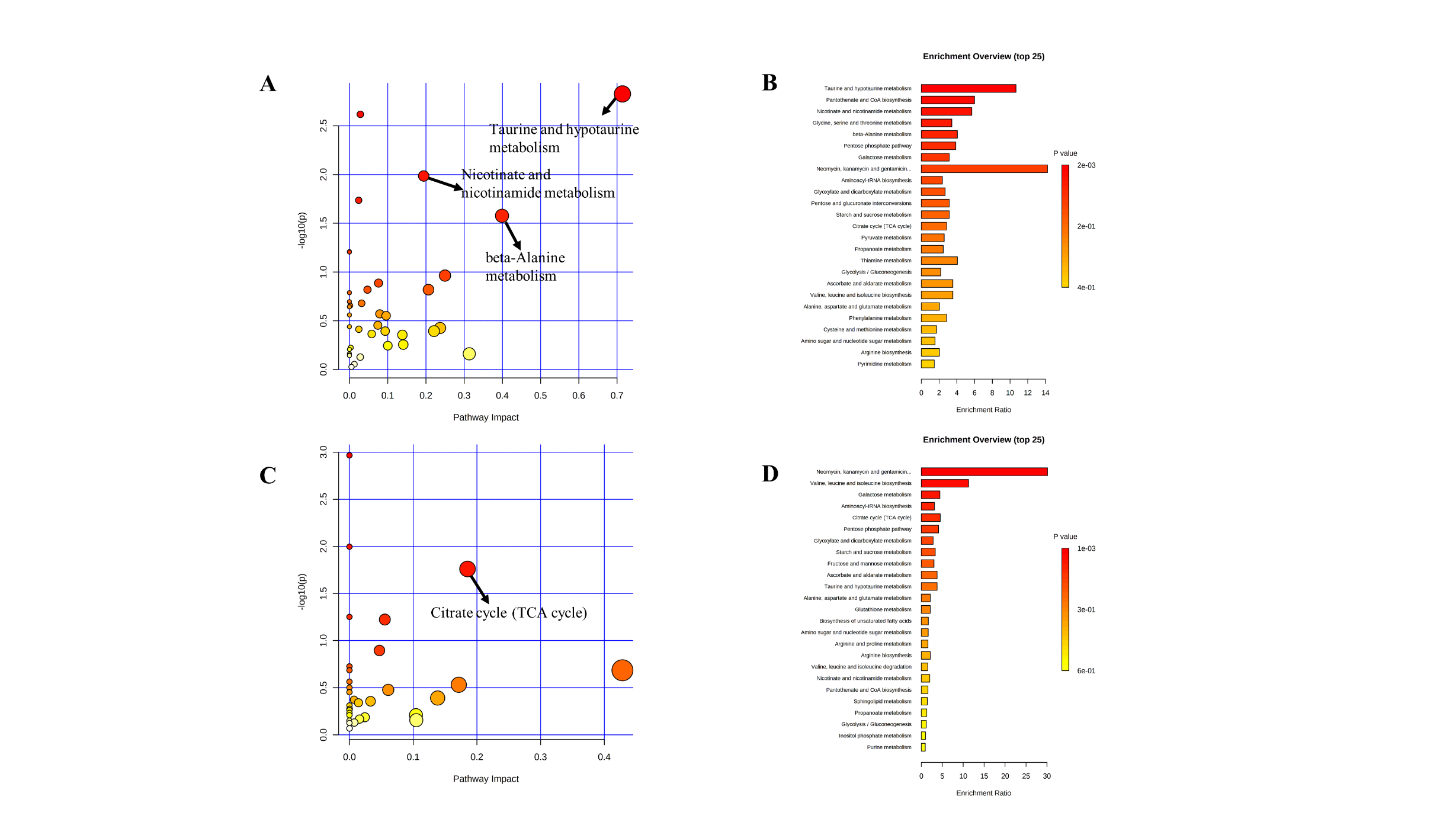
Fig. S6** Significantly changed pathways in serum between the monoglycerides and antibiotic groups on d 5 (**A**) and d 14 (**C**) post-inoculation, respectively. The *x*-axis represents the pathway impact values and the *y*-axis represents the −log(*P*) values from the pathway enrichment analysis. Metabolite set enrichment analysis shows the metabolic pathways were enriched in monoglycerides compared with antibiotic on d 5 (**B**) and d 14 (**D**) post-inoculation, respectively. Both pathway analysis and metabolite set enrichment analysis were performed using identified metabolites with VIP > 1

**
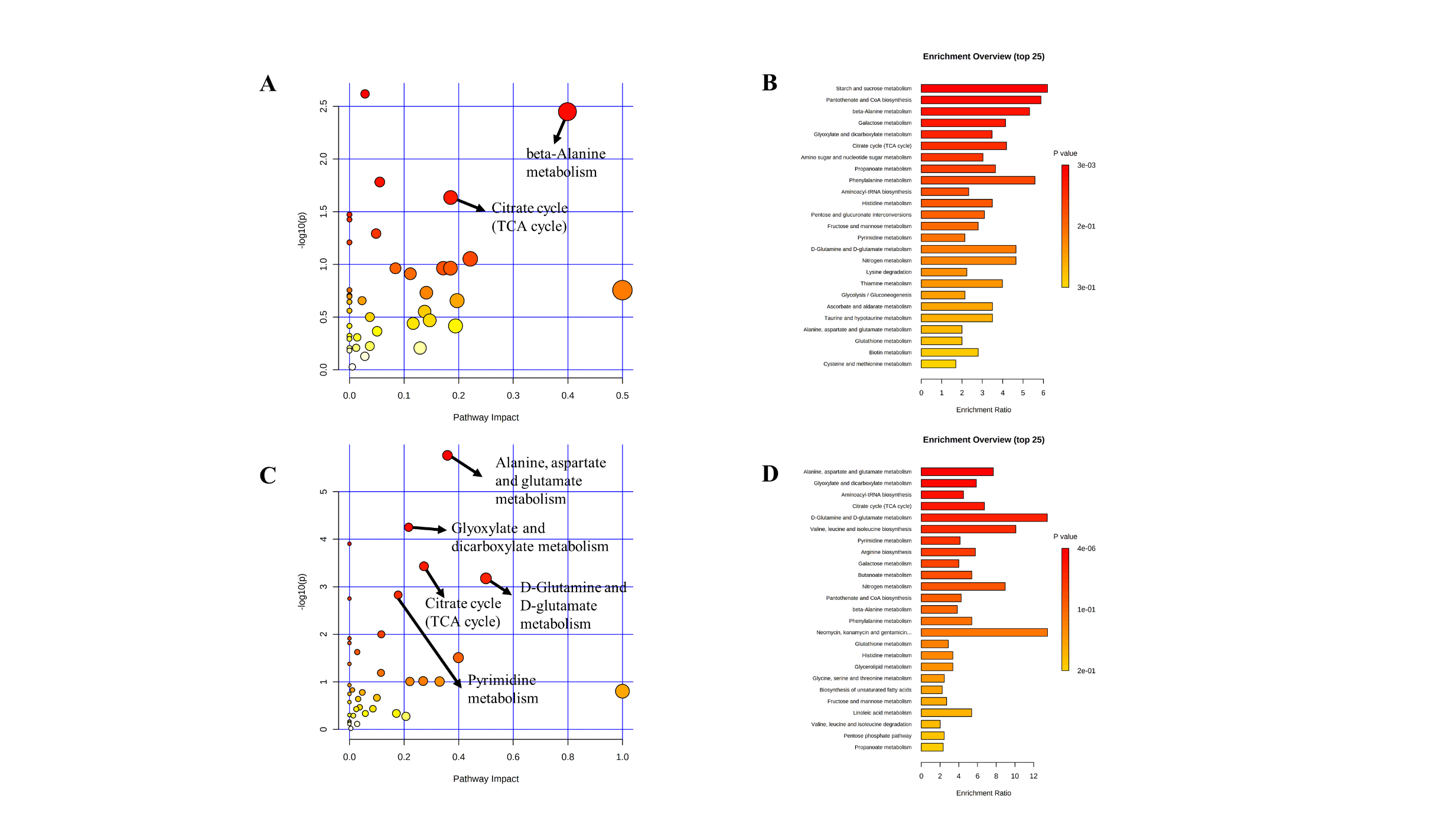
Fig. S7** Significantly changed pathways in serum between the high-dose zinc oxide (ZNO) and antibiotic groups on d 5 (**A**) and d 14 (**C**) post-inoculation, respectively. The *x*-axis represents the pathway impact values and the *y*-axis represents the −log(*P*) values from the pathway enrichment analysis. Metabolite set enrichment analysis shows the metabolic pathways were enriched in ZNO compared with antibiotic on d 5 (**B**) and d 14 (**D**) post-inoculation, respectively. Both pathway analysis and metabolite set enrichment analysis were performed using identified metabolites with VIP > 1
