# Supplementary material for: Effects of monoglyceride blend on systemic and intestinal immune responses, and gut health of weaned pigs experimentally infected with a pathogenic Escherichia coli
Source: J Anim Sci Biotechnol. 2024 Oct 13;15:141. doi: 10.1186/s40104-024-01103-7 (PMC11479547; doi:10.1186/s40104-024-01103-7)
Supplement: Supplementary file 4 — Additional file 4: Fig. S8 Intestinal morphology of enterotoxigenic Escherichia coli F18-challenged weaned pigs fed experimental diets on d 5 post-inoculation. [file 40104_2024_1103_MOESM4_ESM.docx]

**
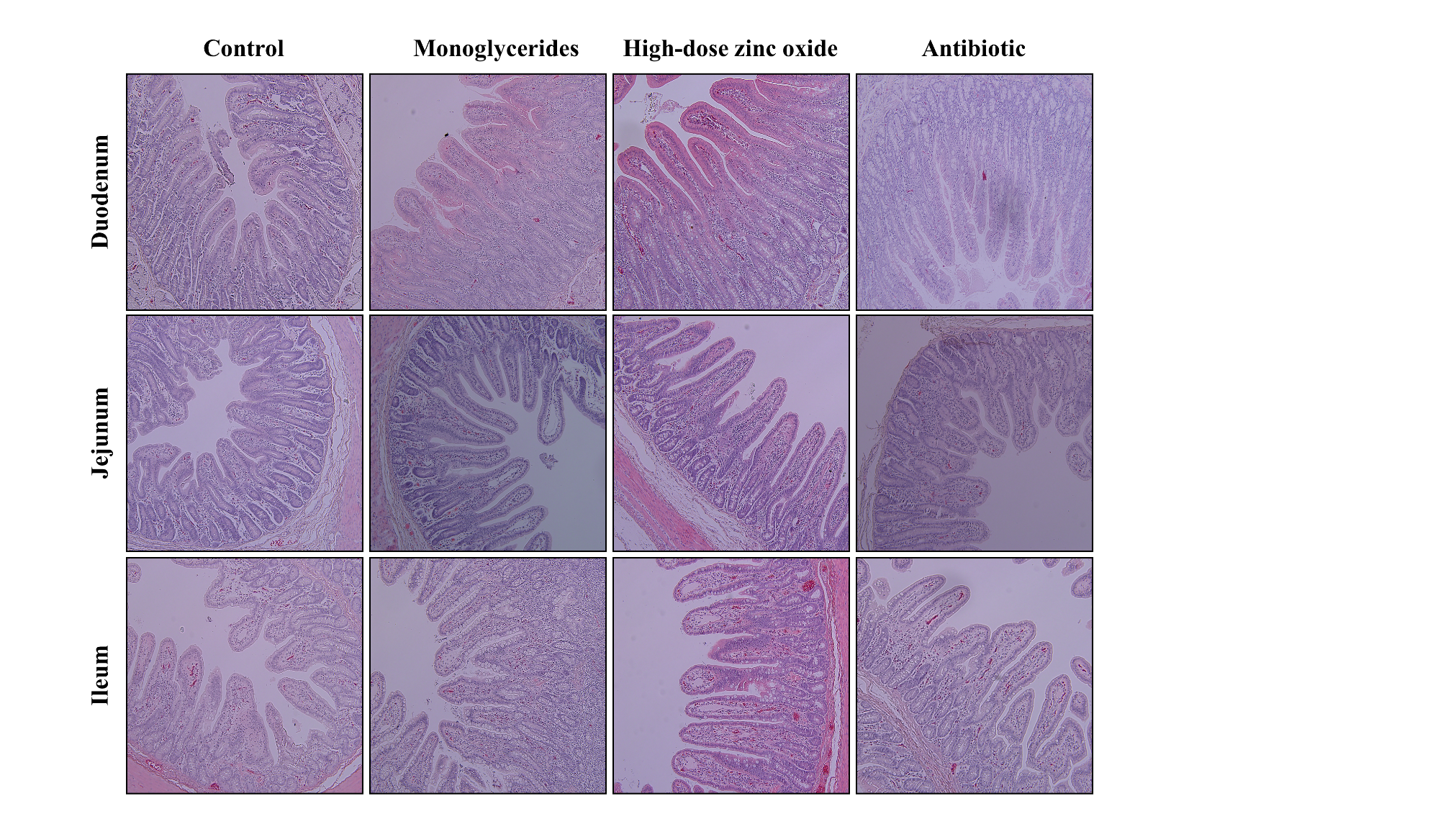
**

**Fig. S8** Intestinal morphology of enterotoxigenic *Escherichia coli* F18-challenged weaned pigs fed experimental diets on d 5 post-inoculation
